# The dietary management of potassium in children with CKD stages 2–5 and on dialysis—clinical practice recommendations from the Pediatric Renal Nutrition Taskforce

**DOI:** 10.1007/s00467-021-04923-1

**Published:** 2021-03-17

**Authors:** An Desloovere, José Renken-Terhaerdt, Jetta Tuokkola, Vanessa Shaw, Larry A. Greenbaum, Dieter Haffner, Caroline Anderson, Christina L. Nelms, Michiel J. S. Oosterveld, Fabio Paglialonga, Nonnie Polderman, Leila Qizalbash, Bradley A. Warady, Rukshana Shroff, Johan Vande Walle

**Affiliations:** 1 University Hospital Ghent, Ghent, Belgium; 2 Wilhelmina Children’s Hospital, University Medical Center Utrecht, Utrecht, The Netherlands; 3 Children’s Hospital and Clinical Nutrition Unit, Internal Medicine and Rehabilitation, University of Helsinki and Helsinki University Hospital, Helsinki, Finland; 4 UCL Great Ormond Street Institute of Child Health, London, UK; 5 University of Plymouth, Plymouth, UK; 6 Emory University, Atlanta, GA USA; 7 Children’s Healthcare of Atlanta, Atlanta, GA USA; 8 Children’s Hospital, Hannover Medical School, Hannover, Germany; 9 University Hospital Southampton NHS Foundation Trust, Southampton, UK; 10 University of Nebraska, Kearney, NE USA; 11 Emma Children’s Hospital, Amsterdam University Medical Center, Amsterdam, The Netherlands; 12 Fondazione IRCCS Ca’ Granda Ospedale Maggiore Policlinico, Milan, Italy; 13 British Columbia Children’s Hospital, Vancouver, Canada; 14 Great Northern Children’s Hospital, Newcastle upon Tyne, UK; 15 Children’s Mercy Kansas City, Kansas City, MO USA

**Keywords:** Potassium, Dietary intake, Chronic kidney disease, Dialysis, Children, Clinical Practice Recommendations (CPRs), Pediatric Renal Nutrition Taskforce (PRNT)

## Abstract

Dyskalemias are often seen in children with chronic kidney disease (CKD). While hyperkalemia is common, with an increasing prevalence as glomerular filtration rate declines, hypokalemia may also occur, particularly in children with renal tubular disorders and those on intensive dialysis regimens. Dietary assessment and adjustment of potassium intake is critically important in children with CKD as hyperkalemia can be life-threatening. Manipulation of dietary potassium can be challenging as it may affect the intake of other nutrients and reduce palatability. The Pediatric Renal Nutrition Taskforce (PRNT), an international team of pediatric renal dietitians and pediatric nephrologists, has developed clinical practice recommendations (CPRs) for the dietary management of potassium in children with CKD stages 2–5 and on dialysis (CKD2–5D). We describe the assessment of dietary potassium intake, requirements for potassium in healthy children, and the dietary management of hypo- and hyperkalemia in children with CKD2–5D. Common potassium containing foods are described and approaches to adjusting potassium intake that can be incorporated into everyday practice discussed. Given the poor quality of evidence available, a Delphi survey was conducted to seek consensus from international experts. Statements with a low grade or those that are opinion-based must be carefully considered and adapted to individual patient needs, based on the clinical judgment of the treating physician and dietitian. These CPRs will be regularly audited and updated by the PRNT.

**Supplementary Information:**

The online version contains supplementary material available at 10.1007/s00467-021-04923-1.

## Introduction

Abnormal serum potassium (K^+^) levels are common in patients with kidney diseases as the kidneys regulate K^+^ excretion in response to dietary intake; over 90% of K^+^ excretion takes place through the kidneys, and the balance between intracellular and extracellular K^+^ levels that affect serum concentrations is also largely controlled by the kidneys. In those with chronic kidney disease (CKD), dyskalemias usually manifest as hyperkalemia and, rarely, as hypokalemia. Hyperkalemia is defined as a serum K^+^ > 5 mmol/L (= 5 mEq/L) in children and adolescents (> 5.5 mmol/L, 5.5 mEq/L in neonates) and hypokalemia as K^+^ < 3.5 mmol/L (= 3.5 mEq/L). Most patients with dyskalemia are asymptomatic, but patients with severe hyperkalemia can have fatal cardiac arrhythmias. Hence, hyperkalemia that is refractory to medical management is a common reason for initiating dialysis. A recent conference proceeding from Kidney Disease Improving Global Outcomes (KDIGO) states that targeting a serum K^+^ level of 4–5 mmol/L is safe in adults [1]. There is a U-shaped relationship between serum K^+^ levels and cardiovascular and kidney outcomes, with increased risk of poor outcomes apparent at relatively mild levels of dyskalemia. The optimal K^+^ serum level in children with CKD is not known.

In CKD patients, the dietary management of K^+^ can be particularly challenging as plant-based diets that are widely considered to be healthy are often high in K^+^. Children with CKD pose unique challenges as the provision of adequate energy, protein, and micronutrients for growth cannot be compromised, and specialized low K^+^ formula, in some children, may not be palatable. In addition, a small group of children with CKD or on dialysis can have persistent hypokalemia, usually as a result of inherited or acquired renal tubular disorders, but sometimes also as a consequence of intensified hemodialysis regimens. Although several studies describe the prevalence of abnormal K^+^ levels as well as associated clinical complications and their medical management, little is known about the dietary requirements and management of K^+^ in children with CKD and on dialysis. There are no high-quality studies on the dietary management of dyskalemias in children with CKD to guide evidence-based practice.

The Pediatric Renal Nutrition Taskforce (PRNT), an international team of specialist renal dietitians and pediatric nephrologists, has developed clinical practice recommendations (CPRs) for the dietary management of K^+^ in children with CKD stages 2–5 and on dialysis (CKD2–5D).These CPRs are designed to provide information and assist in decision-making in order to improve patient outcome. Given the low quality of available evidence, the CPRs are not intended to define a standard of care and may need to be adapted to individual patient needs based on the clinical judgment of the treating physician and dietitian.

## Methods

The composition of the PRNT and the detailed development process for the CPRs, literature search criteria, grading of evidence, and plans for audit and revision of the CPRs have been described [2, 3]. The PICO (Patient, Intervention, Comparator and Outcome) format [4] has been used to develop recommendations that provide specific actionable advice, including choosing between alternative approaches in particular clinical situations.

### PICO terms

Population: Children from birth to 18 years of age with CKD2–5D.

Intervention: Nutritional requirements for K^+^ in children at different stages of CKD and on dialysis.

Comparator: Nutritional requirements for K^+^ in age-matched healthy children or no comparator.

Outcomes: Dietary K^+^ intake that maintains normal serum K^+^ levels in children with CKD2–5D.

#### Literature search

Details on the literature search are described in Supplementary Table 1. Original publications on the K^+^ requirements in healthy children were reviewed and used to help develop CPRs for children with CKD stages 2–5D. There are no randomized controlled trials (RCTs) on K^+^ management in healthy children or those with CKD. All studies are observational, and most are retrospective. Due to the lack of high-quality studies, we have included all studies with findings relevant to outcomes, irrespective of patient numbers or duration of follow-up. In the absence of applicable studies, guidance is based on the opinion of experienced dietitians and nephrologists from the PRNT.

#### Developing CPRs

After critically reviewing the literature for each PICO question, evidence tables were prepared (Supplementary Table 2) and CPRs developed, with a detailed “evidence and rationale” section to support each statement. CPRs were graded as suggested by the American Academy of Pediatrics (Supplementary Table 3) [5]. A Delphi survey (e-questionnaire) was conducted as previously described [2, 3]. It was agreed a priori that at least a 70% level of consensus was required for each statement, failing which the CPR would be discussed in the PRNT group, adapted if required, and reviewed again by the Delphi panel until a consensus level of at least 70% was achieved.

#### Practical application and guideline management

**Fig. 1:**
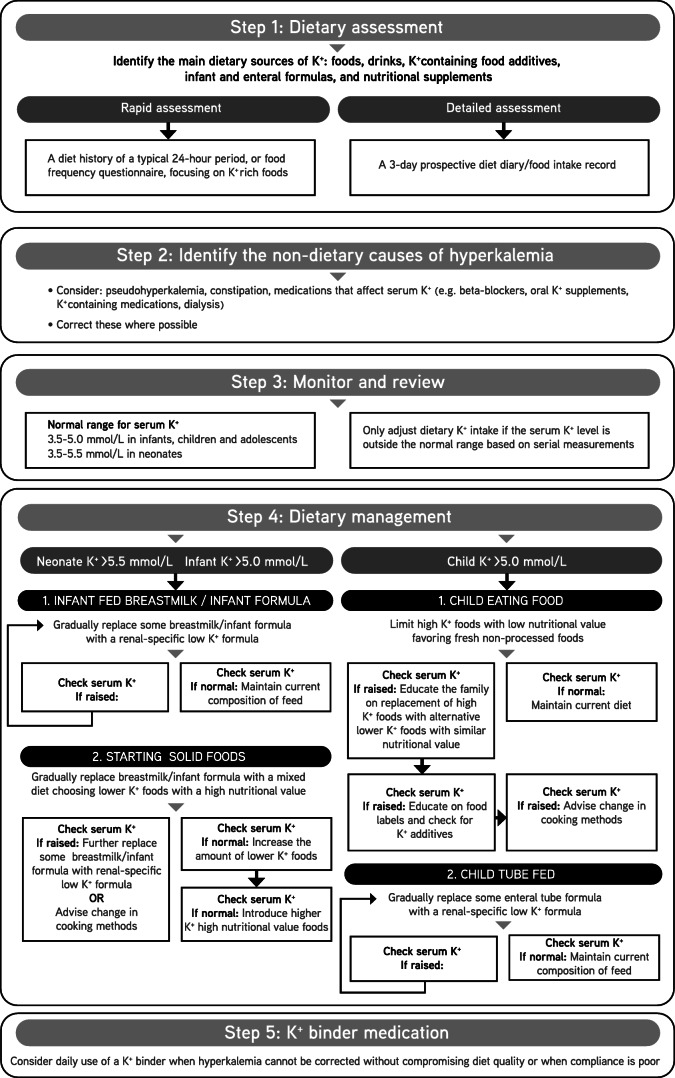
Flowchart summarizing dietary management of hyperkalemia

A flow sheet to guide the management of hyperkalemia in children with CKD has been developed and is shown in Fig. 1. These CPRs will be audited and revised periodically by the PRNT.

### Clinical practice recommendations

1. What are the main dietary sources of potassium for an infant, child and adolescent?
  1.1 The main dietary sources of potassium for infants are breastmilk or infant formula (not graded).
  1.2 The main natural dietary sources of potassium for children and adolescents are milk, potatoes, vegetables, cereals, fruits, and meat (not graded).
  1.3 Food additives that contain potassium salts contribute to potassium intake (D weak).

### Evidence and rationale

#### Main dietary sources of potassium

K^+^ is naturally present in all types of foods. Food preparation techniques, such as shredding, boiling, and microwave cooking, may lead to significant losses in K^+^ content [6]. The contribution of K^+^-rich foods to the diet is geographically varied [7], as illustrated by the surveys in Supplementary Tables 4a, 4b, and 4c which show the contribution of foods to the daily intake of K^+^ for different ages from various countries [6, 8–10]. As the sole source of nutrition for young infants, breastmilk and infant formula are the major sources of dietary K^+^. The percentage contribution of K^+^ intake from milk decreases when solid foods are introduced to the infant’s diet. According to the surveys above, the main sources of K^+^ from 18 months to 18 years are milk and milk products, starchy roots and tubers such as potatoes, vegetables, cereals (grains) and cereal products, fruit and fruit products, and meat. The typical K^+^ content per portion of foods and per 100 g is shown in Tables 1, 2, and 3 (UK data).

**Table 1:** A guide to the potassium content of fruits*

Food	Portion size	Potassium (mg per portion)	Potassium (mg per 100 g)
Fruits (edible weight, medium-sized, and fresh unless stated otherwise)			
High potassium (> 117 mg (3 mmol) per portion)			
Apricot, ready-to-eat, semi-dried	20 g (3)	208	1039
Apricot	80 g (2)	216	270
Avocado	75 g (½)	380	507
Banana	80 g (1 small)	264	330
Dates, raw	30 g (2)	123	410
Fig, ready-to-eat, semi-dried	40 g (2)	314	784
Grapes	60 g (12)	129	215
Kiwi fruit	60 g (1)	173	289
Melon, cantaloupe	150 g (1 slice)	315	210
Nectarine	90 g (1)	231	257
Orange	120 g (1 small)	146	122
Orange juice, chilled	100 ml	158	158
Peach	110 g (1)	176	160
Prunes, ready-to-eat, semi-dried	30 g (4)	220	734
Raisins, dried	30 g (1 Tbsp)	267	889
Strawberries	80 g (7)	136	170
Watermelon	120 g (10 balls)	120	100
Moderate potassium (39–117 mg (1–3 mmol) per portion)			
Apple	100 g (1)	100	100
Blackberries	40 g (8)	62	154
Cherries	40 g (10)	95	238
Clementine, mandarin, satsuma, tangerine	50 g (1 small)	64	128
Grapefruit	80 g (½)	103	129
Mango	75 g (½)	113	150
Passion fruit	30 g (2)	60	200
Pear	100 g (1)	105	105
Pineapple	80 g (1 large slice)	97	121
Plum	55 g (1)	105	190
Raspberries	60 g (15)	98	164
Lower potassium (< 39 mg (1 mmol) per portion)			
Blueberries	45 g (2 Tbsp)	30	66
Olives, no stones	30 g (10)	27	91

The routine omission of fruits from the diet based simply on their K content should be discouraged, considering that the bioavailability of K in unprocessed plant foods is no more than 60% and they offer other nutritional benefits (vitamins, minerals, fiber). It may be beneficial to choose foods with a low K-fiber ratio to enable a higher fiber intake to be maintained while lowering dietary K [11] (Supplementary Table 12)

*Refer to country specific composition tables where possible.

Data sourced and adapted from McCance and Widdowson's Composition of Foods Integrated Dataset (CoFID), Public Health England, 2019 [12]

*Tbsp* rounded tablespoon, *tsp* rounded teaspoon

**Table 2:** A guide to the potassium content of vegetables and pulses*

Food	Portion size	Potassium (mg per portion)	Potassium (mg per 100 g)
Vegetables and pulses (legumes) (edible portion)			
High potassium (> 117 mg (3 mmol) per portion)			
Asparagus, steamed	75 g (3 spears)	213	283
Baked beans, canned in tomato sauce	80 g (2 Tbsp)	218	272
Beetroot, boiled	35 g (1 small)	106	302
Broad beans, boiled	60 g (1 Tbsp)	248	413
Brussels sprouts, boiled	40 g (1 Tbsp)	161	404
Mushrooms, fried in oil	40 g (4 medium)	217	542
Parsnip, boiled	40 g (1 Tbsp)	128	319
Plantain, boiled	50 g (¼ medium)	200	400
Sweet potato, boiled	40 g (1 Tbsp)	150	375
Tomato, raw	65 g (1 small)	145	223
Tomato, canned	100 g (¼ can)	212	212
Yam, boiled	60 g (1 small egg-sized)	162	271
Moderate potassium (39–117 mg (1–3 mmol) per portion)			
Aubergine (eggplant), fried in oil, including skin	65 g (¼ medium)	111	170
Broccoli, boiled	40 g (1 Tbsp)	85	212
Butternut squash, baked	40 g (1 Tbsp)	97	242
Cabbage, green, boiled	40 g (1 Tbsp)	75	187
Carrot, boiled	40 g (1 Tbsp)	66	166
Cauliflower, boiled	40 g (1 Tbsp)	86	215
Chickpeas, dried, boiled	40 g (1 Tbsp)	112	281
Courgette (zucchini), boiled	40 g (1 Tbsp)	95	238
Cucumber, raw	40 g (6 slices)	62	156
Hummus	30 g (1 Tbsp)	57	190
Leek, boiled	40 g (1 Tbsp)	68	169
Lentils, red, split, dried, boiled	40 g (1 Tbsp)	88	220
Okra, boiled	30 g (6 medium)	64	213
Onion, fried in oil	30 g (1 Tbsp)	57	189
Peas, boiled	30 g (1 Tbsp)	69	230
Peppers, red, yellow, raw, sliced	30 g (3 rings)	61	203
Spinach, boiled	40 g (1 Tbsp)	64	230
Swede, boiled	40 g (1 Tbsp)	70	175
Sweetcorn, kernels canned in water, drained	30 g (1 Tbsp)	47	158
Turnip, boiled	40 g (1 Tbsp)	80	200
Lower potassium (< 39 mg (1 mmol) per portion)			
Bean sprouts, raw	20 g (1 Tbsp)	15	74
Peppers, green, raw, sliced	30 g (3 rings)	36	120
Pumpkin, boiled	40 g (1 Tbsp)	34	84
Tofu, steamed	40 g (1 Tbsp)	25	63

The routine omission of vegetable and legumes from the diet based simply on their K content should be discouraged, considering the bioavailability of K in unprocessed plant foods is no more than 60% and they offer other nutritional benefits (vitamins, minerals, fiber). It may be beneficial to choose foods with a low K-fiber ratio to enable a higher fiber intake to be maintained while lowering dietary K [11] (Supplementary Table 12)

*Refer to country specific composition tables where possible.

Data sourced and adapted from McCance and Widdowson's Composition of Foods Integrated Dataset (CoFID), Public Health England, 2019 [12]

*Tbsp* rounded tablespoon, *tsp* rounded teaspoon

**Table 3:** A guide to the potassium content of other food groups*

Food	Portion size	Potassium (mg per portion)	Potassium (mg per 100 g)
Milk and dairy products			
Human breastmilk, mature^	100 ml	58	58
Standard whey dominant infant formula (average)	100 ml	70	70
Cow’s milk, whole	100 ml	157	157
Custard, canned**	100 g (3 Tbsp)	129	129
Fromage frais, fruit flavor	60 g (1 small pot)	86	143
Ice cream, vanilla, soft scoop**	60 g (1 scoop)	98	163
Yogurt, whole milk, fruit	125 g (1 small pot)	213	170
Potatoes			
Potatoes, new, boiled, with skin	60 g (1 small egg-sized)	226	377
Potatoes, old, baked in jacket, flesh only, no skin	100 g (1 small)	360	360
Potatoes, chips, cut fine, fast food**	75 g (small portion)	408	544
Potatoes, old, mashed with butter	45 g (1 Tbsp)	151	337
Potatoes, old, roast	60 g (1 small egg-sized)	358	597
Cereal (grain) and cereal products			
Bread: white, brown, whole meal	40 g (1 thick slice)	54, 86, 101	134, 216, 253
Breakfast cereal, cornflakes, fortified	20 g (3 Tbsp)	18	88
Breakfast cereal, muesli, Swiss style, unfortified	30 g (2 Tbsp)	87	290
Breakfast cereal, porridge made with whole milk, fortified	135 g (3 Tbsp)	268	199
Breakfast cereal, puffed wheat, honey coated, fortified	20 g (3 Tbsp)	38	188
Breakfast cereal, wheat biscuits, fortified	20 g (1 biscuit)	79	397
Cake: sponge with jam and butter cream, chocolate fudge, plain fruit**	60 g (1 slice)	79, 214, 231	132, 357, 385
Cookie (biscuit), digestive, half coated with chocolate**	17 g (1)	44	258
Cookie (biscuit), semi-sweet**	14 g (2)	24	168
Cookie (biscuit), short, sweet**	20 g (2)	31	155
Pasta, white, dried, boiled	90 g (3 Tbsp)	103	114
Rice: white, brown, boiled	80 g (2 Tbsp)	10, 50	12, 62
Nuts and seeds			
Almonds	13 g (6 whole)	95	733
Brazil nuts	10 g (3 whole)	66	660
Cashews, hazel nuts	10 g (10 whole)	72	720
Peanuts, unsalted	13 g (10 whole)	87	670
Peanut butter, smooth	12 g (thinly spread on 1 slice of bread)	84	700
Walnuts	20 g (6 halves)	90	450
Pumpkin seeds	20 g (1 Tbsp)	164	820
Sunflower seeds	16 g (1 Tbsp)	114	710
Tahini paste	19 g (1 tsp)	110	580
Meat, chicken, fish			
Burger, beef, commercial, grilled, average**	35 g (1 patty)	133	380
Chicken, lamb, beef, pork, roasted, meat only	50 g (1 thick slice)	165, 180, 185, 200	330, 360, 370, 400
Chicken nuggets**	70 g (4)	195	278
Cod, steamed/microwaved, flesh only	60 g (one-half medium fillet)	254	424
Cod in batter, baked	60 g (one-half small fillet)	138	230
Salmon, baked/grilled, flesh only	50 g (one-half medium fillet)	206	412
Miscellaneous			
Potato crisps**	25 g (1 small bag)	332	1328
Tortilla chips, corn snacks**	25 g (1 small bag)	71, 82	285, 329
Twiglets**	25 g (1 small bag)	115	460
Chocolate: plain, milk**	50 g (1 small bar)	150, 226	300, 451
Coffee, instant, powder**	2 g (1 tsp)	76	3780
Drinking chocolate, cocoa, powder**	6 g (1 tsp)	30, 90	495, 1500
Yeast extract	1 g (thin scraping on I slice of bread)	21	2100

Data sourced and adapted from McCance and Widdowson’s Composition of Foods Integrated Dataset (CoFID), Public Health England, 2019 [12]

*Tbsp* rounded tablespoon, *tsp* rounded teaspoon

*Refer to country specific composition tables where possible

**When there is a need to reduce potassium intake, foods of low nutritional quality should be targeted first

^Department of Health and Social Security. The composition of mature human milk. Report on Health and Social Subjects No 12, HMSO, London, 1977. More recent analyses of breastmilk may show a different potassium content. For processed foods, check manufacturers’ data

**Table 4:** Summary of recommendations

	Category	Recommendation	Grade
1	Main dietary sources of potassium for children with CKD2–5D	1.1 The main dietary sources of potassium for infants are breastmilk or infant formula.	Not graded
		1.2 The main natural dietary sources of potassium for children and adolescents are milk, potatoes, vegetables, cereals, fruits and meat.	Not graded
		1.3 Food additives that contain potassium salts contribute to potassium intake.	D Weak
2	Assessment of potassium intake for children with CKD2–5D	2.1 A diet history may not give an accurate assessment of potassium intake.	Not graded
		2.2 Assess dietary potassium intake in those with dyskalemia.	D Weak
		2.3 A diet history of a typical 24-hour period, or food frequency questionnaire, focusing on potassium-rich foods, can identify the main dietary sources of potassium.	D Weak
3	Potassium requirements for children with CKD2–5D	3.1 Potassium requirements are based on the level of kidney function, weight, growth, renal potassium losses, extra-renal potassium losses, clearance by dialysis, and medications that may increase or decrease serum potassium levels.	D Weak
		3.2 Adjust the dietary potassium intake based on serum potassium levels, aiming to maintain potassium levels within the normal range.	D Weak
4	Management of dyskalemia due to non-dietary causes	4.1 Correct the non-dietary causes of dyskalemia, and adjust the dialysis prescription where appropriate, before adjusting the dietary potassium intake.	C Moderate
5	Management of hyperkalemia in children with CKD2–5D	5.1 Severe, life-threatening hyperkalemia requires rapid medical intervention and discontinuation of all sources of potassium from medications, parenteral fluids, formulas and diet.	X Strong
		5.2 In a child with persistent or recurrent episodes of hyperkalemia, decrease the intake of potassium without compromising nutrition to maintain the serum potassium within the normal range.	C Weak
		5.2.1 For infants receiving breastmilk, reduce potassium intake by substituting some of the breastmilk with a renal-specific low potassium infant formula.	D Weak
		5.2.2 For children receiving formula or enteral tube feed, reduce potassium intake by combining standard formula stepwise with a renal-specific low potassium formula.	D Weak
		5.2.3 If a renal-specific low potassium formula is not available, use of a potassium-binding resin and decanting of the formula may be considered. Monitor other electrolytes that may be altered by the potassium binder.	C Weak
		5.2.4 For children who are eating, avoid foods containing potassium additives in the first instance.	D Weak
		5.2.5 If hyperkalemia persists, decrease potassium intake by reducing high potassium foods, particularly those with a low nutritional value.	D weak
		5.2.6 Advise parents and caregivers on food preparation techniques that reduce the potassium content.	C Weak
		5.2.7 The daily use of an oral potassium binder to control serum potassium level may be considered when hyperkalemia cannot be corrected without compromising diet quality, or when dietary compliance is poor.	D Weak
6	Management of hypokalemia in children with CKD2–5D	6.1 Severe, life-threatening hypokalemia requires prompt medical intervention, usually requiring intravenous potassium infusion.	X Strong
		6.2 In a child with persistent hypokalemia, increase the dietary potassium intake, targeting foods with high nutritional quality, to maintain serum potassium within normal range.	D Weak
		6.3 If applicable, review and adjust potassium lowering medications and the dialysis prescription.	C Moderate

#### Food additives

Regulations for the use of K^+^-containing food additives differs between countries. In Europe, it is mandatory for K^+^-containing additives to be listed on food packaging by E-number or by name [13] (Supplementary Table 5), however only their presence can be verified from food labels as no quantitative data is provided. The USA will have such mandatory food labelling from July 2021 [14], and Canada is undergoing a transition period due for completion in 2022 [15]. Without such regulations, the addition of K^+^ salts to a food may be unknown. A food described as “enhanced” may have K^+^-containing additives [16].

The use of K^+^-containing additives is increasing [16, 17], in particular to reduce the salt content of food products where sodium chloride is replaced with K^+^ chloride [18]. These additives can more than double the K^+^ content of a food, e.g. from 325 to 900 mg/100 g in meat, poultry and fish products [19]. If the majority of preferred foods in a child’s diet are processed “ready-to-eat,” then the K^+^ intake will be higher than if fresh foods were consumed. Of note, foods with added K^+^ salts may also contain phosphate-based additives, e.g., processed meats and cheeses, and baked goods made with flour. If K^+^ chloride, as a substitute for table salt, is used by other members of the household, the family must be cautioned against its use in the child with CKD.

#### Bioavailability

A few small studies in adults have examined the bioavailability of K^+^, with urinary K^+^ used to quantify absorption since almost all absorbed K^+^ is excreted in the urine [17, 20–22]. In a small cross-over feeding trial [20], bioavailability of K^+^ from unprocessed fruits and vegetables was lower than from animal food and fruit juices, with bioavailability from unprocessed plant foods being no more than 60%. The K^+^ in plant foods is mostly intracellular, and since plant cells are not easily digested, the K^+^ is excreted in feces. A similar bioavailability of 50–60% was found in two small studies of the DASH (Dietary Approach to Stop Hypertension) diet in adults with CKD [21, 22]. Considering the bioavailability of K^+^ from fruits and vegetables, they should not be routinely omitted from the diet based simply on their K^+^ content.

In contrast, the bioavailability of K^+^ from processed foods with K^+^-containing additives was much greater at 90–100% [23, 24]. Hence, in children with hyperkalemia, foods containing K^+^ additives must be avoided in the first instance before restricting fresh foods. As the intake of processed foods can be a hidden source of extra K^+^, there should be careful education for patients and caregivers about the reading of packaging labels [16].

The “Mediterranean” diet for patients with CKD, as described by Chauveau et al. [25], favors natural foods over processed foods along with a high intake of fruits, vegetables and wholegrain cereals; the diet has the advantage of being both low in food additives and rich in high fiber plant-based foods.

2. How is potassium intake assessed in children with CKD 2–5D?
  2.1. A diet history may not give an accurate assessment of potassium intake (not graded).
  2.2. Assess dietary potassium intake in those with dyskalemia (D weak).
  2.3. A diet history of a typical 24-h period, or food frequency questionnaire, focusing on potassium-rich foods, can identify the main dietary sources of potassium (D weak).

### Evidence and rationale

The dietary K^+^ intake does not need to be modified unless the child exhibits dyskalemia, whether acute or chronic. Quantifying the exact K^+^ intake is not possible unless the child is exclusively fed with formula, either orally or by enteral tube feed. A lack of data on the bioavailability of K^+^ from foods, missing data on labels of processed “ready-to-eat” foods containing K^+^ salts as food additives, and unknown losses in food preparation make it impossible to calculate the exact amount of K^+^ in the diet. Although there may be country-specific food labelling regulations for the presence of K^+^-containing additives in processed foods, there is no requirement for quantitative data. Identifying the main dietary sources of K^+^, as well as taking into account variable amounts from potential “hidden” sources from additives, is suggested.

A retrospective diet recall of a typical 24-h period can be used to rapidly identify the main dietary sources of K^+^, including K^+^-containing additives. Food frequency questionnaires may also be helpful to gather information on eating patterns and can be targeted to the consumption of high K^+^ foods. A 3-day prospective diet diary/food intake record may be used when more detailed information is required. An estimate of the total K^+^ sources should consider contributions from diet, infant and enteral formulas, nutritional supplements, and medications (Supplementary Table 6).

3. What are the potassium requirements for infants, children and adolescents with CKD2–5D?
  3.1. Potassium requirements are based on the level of kidney function, weight, growth, renal potassium losses, extra-renal potassium losses, clearance by dialysis, and medications that may increase or decrease serum potassium levels (D weak).
  3.2. Adjust the dietary potassium intake based on serum potassium levels, aiming to maintain potassium levels within the normal range (D weak).

### Evidence and rationale

#### Healthy children

A normal serum K^+^ level is necessary for multiple cellular functions. In healthy children, a normal serum K^+^ is maintained despite wide variations in K^+^ intake due to homeostatic mechanisms that function largely by adjusting urinary excretion of K^+^. Moreover, the presence of K^+^ in most foods ensures an adequate intake to maintain a normal serum K level except if there is severe energy (caloric) deprivation or an extremely restricted diet. There is evidence in adults that a low K^+^ intake is associated with a variety of adverse clinical consequences, including hypertension, stroke, and cardiovascular disease [26–28]. Similar evidence is limited in children.

The recommended K^+^ intakes for healthy populations of children of different ages are presented in Supplementary Table 7. A variety of approaches have been used by national and international organizations to estimate K^+^ requirements in healthy children (e.g. based on normal dietary intake, median breastmilk intake, dietary surveys, extrapolation from adult data). However, there is very little evidence to support any of these published recommendations.

#### Children with CKD 2–5D

K^+^ requirements in children with CKD are likely to vary tremendously based on level of kidney function, weight, growth, renal K^+^ losses, presence of acidosis, extra-renal K^+^ losses, clearance by dialysis, and the use of medications that may increase or decrease K^+^ levels. There is no data providing minimum or maximum K^+^ requirements for children with CKD. In adults with hyperkalemia and CKD, data suggests that a K^+^ intake less than 2000 to 3000 mg/day (50 to 75 mmol/day) or 1 to 1.3 mmol/kg/day will maintain a normal serum K^+^ concentration in most patients [29, 30]. Extrapolating from this limited adult data, KDOQI recommends an initial target intake of 1 to 3 mmol/kg/day for infants and young children [31].

Since the publication of the KDOQI guidelines, there are no new studies on the appropriate K^+^ intake of children with CKD. There are, however, some studies describing K^+^ intake of children with CKD and their dietary sources or correlation with diet quality, but not with the effect on serum K^+^ levels [32, 33]. A cross-sectional study in North American children with CKD2–3b (estimated glomerular filtration rate (eGFR) of 30–90 ml/min/1.73 m^2^) demonstrated that the K^+^ intake increased with age, with mean intakes of 2084, 2377, 2334, and 2991 mg/day respectively for ages 1–3 years, 4–8 years, 9–13 years, and 14–18 years [32]. In a cross-sectional study in the USA and Canada, the daily K^+^ intake decreased as CKD advanced due to reduced appetite and more dietetic counselling [33]. The mean K^+^ intake was 66 mg/kg/day (1.7 mmol/kg/day), which is within the KDOQI range (1–3 mmol/kg) and similar to trends in the general population. The major dietary sources of K^+^ were milk, fruit, fast foods, fruit juice, and potatoes. However, more than one-third of children did not receive dietetic counselling and 7% had hyperkalemia. In another study in children on dialysis, intake of potassium was 44 mg/kg/day (1.1 mmol/kg/day) in anuric patients and 61 mg/kg/day (1.5 mmol/kg/day) in patients with residual urine output [34].

There are no recommendations for the K^+^ requirement of infants and children with CKD due to a lack of data. In general, for infants and young children, 1–3 mmol/kg/day may be a reasonable place to start [31].

When determining K^+^ intake in children with CKD, it is important to consider trends in serum K^+^ levels rather than single values before any dietary adjustments are made. Difficulties with blood sampling can cause cell hemolysis and release of intracellular K^+^, giving rise to spuriously elevated serum K^+^ levels or pseudohyperkalemia; this is especially common in infants.

4. Management of dyskalemia due to non-dietary causes
  4.1. Correct the non-dietary causes of dyskalemia, and adjust the dialysis prescription where appropriate, before adjusting the dietary potassium intake (C moderate).

### Evidence and rationale

Hyperkalemia can result from non-dietary causes [35, 36] (Supplementary Table 8). Pseudohyperkalemia, due to hemolysis, fist clenching or trauma during venipuncture, is especially common in infants. Hence, a repeat serum K^+^ level is commonly performed if there is evidence of hemolysis in the sample or the serum K^+^ level is severely elevated or seems inconsistent with the clinical situation. Although K^+^ excretion by the kidneys is not significantly affected until the GFR is less than 15 to 20 mL/min/1.73 m^2^ [26], and decreased GFR is the main risk factor for hyperkalemia in children with CKD, other potential etiologies must be considered.

While oral K^+^ supplements should clearly be reduced or stopped in patients with hyperkalemia, other medications may also cause hyperkalemia. Medications that commonly cause hyperkalemia in children with CKD by decreasing renal K^+^ excretion are renin-angiotensin-aldosterone system inhibitors (including angiotensin converting enzyme inhibitors and angiotensin receptor blockers), calcineurin inhibitors, and K^+^-sparing diuretics. Beta-blockers cause hyperkalemia by decreasing cellular entry of K^+^. In some patients, medications may be stopped or the dose reduced, with a subsequent decrease in the serum K^+^ concentration. In children on dialysis, adjust the dialysis prescription to increase K^+^ clearance if appropriate.

Chronic metabolic acidosis can cause K^+^ shifts from the intracellular to the extracellular space and may also decrease urinary K^+^ excretion and contribute to hyperkalemia. This may be corrected by administration of base supplements. Renal tubular disorders may impair renal K^+^ excretion. Some patients who have decreased aldosterone levels may respond to an oral mineralocorticoid. Constipation may decrease gastrointestinal losses of K^+^, especially in advanced CKD [37].

There are multiple potential non-dietary causes of hypokalemia in children with CKD (Supplementary Table 9). Dialysis, particularly frequent daily or nocturnal hemodialysis, may lead to excessive K^+^ losses and lead to hypokalemia. This may be corrected by adjusting the dialysis prescription, and although this allows many patients to enjoy an unrestricted dietary K^+^ intake, some may require K^+^ supplements. Medications, such as loop or thiazide diuretics, may increase renal losses of K^+^. K^+^-binding resins (e.g. sodium polystyrene sulfonate), prescribed to treat hyperkalemia, may precipitate hypokalemia. Stopping or decreasing the doses of these medications may correct the hypokalemia, although reducing diuretics may not be tolerated in some patients. Such patients may benefit from a K^+^-sparing diuretic to decrease urinary losses or K^+^ supplements. Gastric fluid losses (emesis or via gastrostomy tube) and diarrhea may cause hypokalemia. It is optimal to correct the underlying disorder, but patients may require additional K^+^ intake until the gastric losses decrease. Renal tubular disorders (e.g. cystinosis and Bartter syndrome) may lead to excessive urinary losses of K^+^, even in the setting of advanced CKD. These patients commonly require K^+^ supplements, but they may cause gastric irritation.

5. Management of hyperkalemia in children with CKD2–5D
  5.1. Severe, life-threatening hyperkalemia requires rapid medical intervention and discontinuation of all sources of potassium from medications, parenteral fluids, formulas, and diet (X strong).
  5.2. In a child with persistent or recurrent episodes of hyperkalemia, decrease the intake of potassium without compromising nutrition to maintain the serum potassium within the normal range (C weak).
    5.2.1. For infants receiving breastmilk, reduce potassium intake by substituting some of the breastmilk with a renal-specific low potassium infant formula (D weak).
    5.2.2. For children receiving formula or enteral tube feed, reduce potassium intake by combining standard formula stepwise with a renal-specific low potassium formula (D weak).
    5.2.3. If a renal-specific low potassium formula is not available, use of a potassium-binding resin and decanting of the formula may be considered. Monitor electrolytes and micronutrients that may be altered by the potassium binder (C weak).
    5.2.4. For children who are eating, avoid foods containing potassium additives in the first instance (D weak).
    5.2.5. If hyperkalemia persists, decrease potassium intake by reducing high potassium foods, particularly those with a low nutritional value (D weak).
    5.2.6. Advise parents and caregivers on food preparation techniques that reduce the potassium content (C weak).
    5.2.7. The daily use of an oral potassium binder to control serum potassium level may be considered when hyperkalemia cannot be corrected without compromising diet quality or when dietary compliance is poor (D weak).

### Evidence and rationale

Severe hyperkalemia can cause fatal cardiac arrhythmias and requires rapid medical intervention [37] and is beyond the scope of this guideline.

In children with persistent mild to moderate hyperkalemia, a diet history to identify sources of K^+^ (see statement 2 above), as well as identifying any non-dietary causes of hyperkalemia, is required. Before manipulating the dietary K^+^ content, it is important to ensure sufficient energy intake; deficient energy intake may manifest as hyperkalemia due to catabolism as K^+^ is released due to tissue breakdown [31, 36]. An unexpectedly low urea level for the severity of CKD may indicate deficient nutritional intake and should alert the clinician to undertake a detailed dietary assessment and anthropometry. If increasing energy intake with normal foods is not possible, addition of energy modules to the infant or child’s usual formula or diet can be advised. Most have a low K^+^ content as they contain only glucose polymers, fats, or a combination of the two. Once these factors have been considered, the dietary K^+^ intake can be reduced as described below.

### Managing intake of potassium in infants and children receiving breastmilk and formulas

#### Breastmilk, infant formula, enteral tube formula, and low potassium formulas

Breastfeeding is always the preferred feeding choice for an infant*.* Breastmilk has a low K^+^ content, 73–84 mg/100 kcal [12, 38, 39] compared with the regulated range allowed for standard whey-dominant infant formulas in Europe, and in Canada and the USA (80–160, 80–200 mg/100 kcal, respectively). For some infants and children with CKD, normal amounts of breastmilk, standard whey-dominant infant formula or pediatric enteral tube formula can aggravate hyperkalemia [31]. In order to lower the K^+^ intake, a renal-specific low K^+^ formula (available as infant formula (23 mg K^+^/100 kcal) and pediatric enteral tube formula (18 mg K^+^/100 kcal)) can be used in combination with the usual feed, with due attention to the ensuing changes in nutrient profile, usually decreased calcium and phosphate content and, for some products, an increased sodium content due to the renal-specific formula. While the use of renal-specific formulas is the preferred option to lower K^+^ intake, they are not readily available in all countries. In this case, an approach to reduce K^+^ intake is to dilute standard infant or pediatric enteral formula. However, this must be undertaken with great caution as dilution also reduces the energy, protein, vitamin, and mineral content of the formula. Energy and protein modules must be added to the diluted formula, together with a suitable vitamin and mineral preparation, to restore its full composition and maintain nutritional adequacy. An example of how to achieve this is given in Supplementary Table 10. It is inadvisable to dilute breastmilk and simply add energy modules to restore the energy content; there will also be a dilution of protein, vitamins and minerals, all of which must be replaced to maintain nutritional adequacy. There is no evidence that reducing maternal dietary K^+^ has any impact on the K^+^ content of breastmilk.

The sole use of renal-specific formulas should only be in the short term (hours rather than days) as their low K^+^ content may cause a rapid fall in serum K^+^. They may be used solely in the initial treatment of moderate to severe hyperkalemia, with careful monitoring of the serum K^+^ levels, with the introduction of a standard formula or breastmilk as soon as serum K^+^ levels allow. Some children may benefit from the extended use of a renal-specific formula, but caution is advised as in addition to a decreased K^+^ intake, there will be decreased calcium and phosphate intakes and, with some formulas, an increased sodium intake. Renal-specific low K^+^ liquid oral supplements can be used as an additional source of nutrition to complement normal food intake, but they are not nutritionally complete, are not intended to be used as a sole source of nutrition, and are not available in every country.

#### Pretreatment of breastmilk and formulas to lower potassium content

Pretreatment of expressed breastmilk, infant or enteral formulas and other milk products with sodium polystyrene sulfonate (SPS) or calcium polystyrene sulfonate (CPS) may be an option. Three retrospective studies in infants have shown a reduction in serum K^+^ levels with pretreatment of formula or breastmilk with SPS [40–42]. However, the reduction in K^+^ is variable. As SPS and CPS act as exchange resins, their use may lead to unwanted effects on other minerals. SPS has been shown to increase serum sodium, aluminum, iron, sulfur and pH and decrease calcium, zinc, copper, phosphorus, manganese and magnesium [40, 43–48]. The use of SPS has also resulted in hypokalemia, hypernatremia and hypocalcemia in infants [40]. Pretreatment of formula with CPS may decrease its K^+^ content, but increases the calcium content [45, 47]. Pretreatment with patiromer (a new calcium-based cation exchange polymer) decreases the K^+^ concentration of infant formula. An increase in calcium, magnesium, sodium, and phosphorus content was seen [49]. Pretreatment of formulas (Supplementary Table 11) should be advised by a renal dietitian or an otherwise suitably trained professional.

### Managing potassium in the diet from foods

Randomized studies demonstrating that serum K^+^ can be reduced by adjusting the K^+^ intake from foods are lacking [1]. However, a reduced K^+^ diet is a widely practiced treatment for chronic hyperkalemia [31].

#### Complementary feeding (weaning) in infants

The introduction of solid foods to the infant’s diet varies widely but often begins with vegetables, potatoes, and fruits. These foods have a high K^+^ content, which may potentially aggravate hyperkalemia. Given the high risk for cardiovascular disease in CKD in later life and the development of taste preferences and eating habits in early childhood, a diet rich in whole grains, vegetables, and fruits is desirable [11]. Complementary foods with a lower K^+^ content can be offered, if necessary, by choosing fruits, vegetables and tuberous roots with low or moderate K^+^ content as described in Tables 1, 2, and 3, or by altering cooking methods. Refined cereal products are lower in K^+^ than those made with the whole grain, but the bioavailability of K^+^ may be higher; they are also lower in other essential nutrients and fiber, so they may not be the preferred choice. The use of renal-specific low K^+^ formulas instead of standard infant formulas or later, cow’s milk, allows for inclusion and greater variety of K^+^-containing fruits and vegetables. Meat is a high natural source of K^+^; the amount that can be given in the diet is usually determined by the infant’s protein needs.

#### Childhood

For the older child with hyperkalemia, cautious limitation of high K^+^ foods is warranted, recognizing the risk of compromising adequate energy intake or specific nutrients. Initial interventions should focus on food and drinks with low nutritional value, including potato crisps, chips, chocolate, coffee, custards and ice cream made from cow’s milk or soy, and fruit juices with high K^+^ content (Tables 1, 2, and 3). However, it may be necessary to limit high K^+^-containing foods with high nutritional value. These may include milk and milk products, meat, poultry, fish and high K^+^ fruits and vegetables. Standard infant formula or follow-on formula (designed for infants over 6 months of age) can continue to be given to the young child rather than introducing cow’s milk, as usual, at 1 year of age. For older children, plant-based drinks low in K^+^, such as oat “milk,” may be given. However, rice drinks should be avoided in infants and young children due to their high arsenic content. Alternatively, juice with a low fruit or berry content is an alternative low K^+^ beverage. Yogurts and desserts based on plant protein are lower in K^+^ than those based on cow’s milk. If available and affordable, renal-specific low K^+^ formulas can be continued beyond the age of 1 year. If available, a renal-specific low K^+^ pediatric liquid oral supplement (sip feed) may be used as an aid to manage K^+^ intake while also ensuring a good source of energy and nutrients. Although not a first choice, an adult renal-specific liquid oral supplement may be considered, even in infants [50], although the micronutrient content may not be appropriate for young children.

#### Demineralization of foods by cooking methods

Cooking potatoes and other tuberous roots and legumes in ample water reduces their K^+^ content by 35–80% depending on the food matrix and preparation method, while soaking raw food has very little effect [46, 51–62]. Cooking shredded potatoes reduces K^+^ content more than cooking diced potatoes [53], while double-cooking (bringing the water to the boil and then replacing it with fresh water) reduces K^+^ more than cooking once [54, 55]. Soaking after boiling may further reduce K^+^ content [59]. Compared with boiling, sous vide cooking (low temperature cooking under vacuum) increases the K^+^ content of foods [63]. Frying also increases K^+^ content [59]. Microwave cooking reduces K^+^ content, but to a lesser extent than boiling [61]. The addition of SPS to soaking or boiling water does not further increase K^+^ loss in solid foods [46]. While boiling reduces the K^+^ content of foods, boiling also reduces the amounts of other minerals and water-soluble vitamins in various proportions. Therefore, to ensure nutritional adequacy, this demineralization of foodstuffs should be done on the advice of a renal dietitian or an otherwise suitably trained professional.

#### Salt substitutes and potassium-containing additives

Salt substitutes, recommended for patients with hypertension to reduce sodium intake, are often high in K^+^, content ranging from 200 to 300 mg per 1 g of salt [11] and, therefore, they should not be used by patients with hyperkalemia. In addition, K^+^-containing additives can remarkably increase the K^+^ content of food [19], as described above.

#### Plant-based diets and potassium-fiber ratio

KDIGO suggests a diet promoting low K^+^ plant-based foods, with an emphasis on an overall healthy dietary pattern [1]. In adults, a Mediterranean diet is suggested to improve or prevent the development of chronic diseases, such as cardiovascular disease [11, 25]. Cupisti et al. suggest that high K^+^ foods be classified in relation to their fiber content; choosing foods with a low K^+^-fiber ratio enables a beneficial higher fiber intake to be maintained while lowering dietary K^+^ [11]. The K^+^ content of some foods normalized for unit of fiber is shown in Supplementary Table 12. There is some evidence in adults that increasing dietary fruits and vegetables corrects metabolic acidosis without changing serum K^+^ levels [64, 65] because a plant-based diet also has a high alkali content. The prevention or correction of metabolic acidosis, as well as constipation, may counteract the hyperkalemia-inducing effects of a high K^+^ intake.

We cannot recommend these dietary strategies for children with CKD as there are no studies showing the effect of a Mediterranean or plant-based diet on K^+^ serum levels, but they may be of relevance in future management.

#### Potassium binders

Long-term adherence with a low K^+^ diet can be challenging for many children with stage 5 CKD. Moreover, an unintended consequence of a K^+^-restricted diet may be a shift toward lower dietary quality. Oral K^+^ binders bind K^+^ in the colon, reducing its absorption and increasing fecal excretion [66]. Randomized trials in adults on dialysis have shown that chronic hyperkalemia can be alleviated with durations of up to 1 year for the newer agents, patiromer [67] and sodium zirconium cyclosilicate [68], with less compelling evidence from short-term studies (up to a week) for sodium polystyrene sulfonate [69, 70]. Relatively common and potentially clinically relevant adverse events reported for patiromer include constipation and hypomagnesemia [71] and for sodium zirconium cyclosilicate include edema (5 g of zirconium cyclosilicate contains 400 mg of sodium [68]). These medications have not been approved for use in children as yet, but pediatric clinical trials are in progress.

Sodium (or calcium) polystyrene sulfonate [69, 70], although highly effective at lowering serum K^+^, carries a high risk of causing severe constipation, bowel necrosis, and is poorly tolerated and rarely used for the long-term management of hyperkalemia [72].

6. Management of hypokalemia in children with CKD2–5D
  6.1. Severe, life-threatening hypokalemia requires prompt medical intervention, usually requiring intravenous potassium infusion (X strong).
  6.2. In a child with persistent hypokalemia, increase the dietary potassium intake, targeting foods with high nutritional quality, to maintain serum potassium within the normal range (D weak).
  6.3. If applicable, review and adjust potassium lowering medications and the dialysis prescription (C moderate).

### Evidence and rationale

Severe hypokalemia may cause muscle cramping, muscle weakness, and potentially paralysis and arrhythmias, the latter especially in children with underlying cardiac disease. Rhabdomyolysis may also occur, with the risk increased by exercise. Hypokalemia may slow gastrointestinal motility, leading to constipation, and impair bladder function, which may cause urinary retention [30]. Severe hypokalemia, especially if symptomatic, is treated with intravenous K^+^, but enteral K^+^ supplements and measures to decrease K^+^ losses (see below) should also be implemented if possible.

Management of chronic hypokalemia may include dietary changes and adjustment in the medical management. If a patient is receiving a K^+^ binder, it should be stopped or the dose reduced. Additional interventions are only needed once the K^+^ binder is stopped. In some cases, if medically acceptable, other medications that cause K^+^ wasting, most commonly loop or thiazide diuretics, may be reduced or stopped. If possible, medical causes of hypokalemia should be addressed (e.g. diarrhea). In some patients, K^+^-sparing diuretics may be utilized to reduce urinary losses of K^+^. Similarly, adjustments in dialysis may decrease K^+^ losses. Oral K^+^ supplements are utilized in patients who do not respond to dietary changes or other medical interventions.

Dietary adjustments may be very effective at addressing hypokalemia, especially in patients who are receiving a K^+^-restricted diet. In children receiving formula or enteral supplements, the K^+^ content may be increased. The targeted increase in K^+^ delivery depends on the severity of the hypokalemia and the clinical situation. Reasonable increases are 0.5–1 mmol/kg per day, with a maximum of approximately 50 mmol per day. However, formula compositions may limit the possible adjustments. In addition, it is important to consider the effect on the intake of other nutrients (e.g. phosphorus). It may take 3–7 days to appreciate the full effect of any change of intake on the serum K^+^. K^+^ supplements may be added to formula if changing the formula is not possible or effective.

### Results of the Delphi survey

The Delphi survey was sent to 39 pediatric nephrologists and 27 dietitians from 22 countries. Of these, 47 returned a completed survey, a 71% overall response rate. The names of all respondents are listed under “Acknowledgments” below. Of the 20 clinical practice recommendation statements overall, an 89% consensus was achieved with a “strongly agree” or “agree” response and an 8% “neutral” response. The Delphi responses reflected the wide variations in practice that can be expected in the absence of robust evidence, and none of the responses was based on published studies.

Three statements received a “disagree” response, with the highest disagree rate being 23% in response to statements 2.1 and 2.2. To clearly state that is it not possible to accurately assess dietary K^+^ intake, we have added in a new statement, with further explanation under the “evidence and rationale” section. There was also some disagreement about the management of dyskalemia in breastfed infants, statement 5.2.1. The lack of renal-specific low K^+^ formulas in some countries may lead to adaptations in practice that cannot be accounted for in this CPR. Further clarifications to the text have been provided as suggested by the respondents.

### Summary of recommendations

A summary of recommendations is provided in Table 4.

## Research recommendations

1. To investigate the effectiveness of 24-h dietary recall compared with a 3-day diet diary (semi-quantitative or weighed) or a food frequency questionnaire as a tool to assess the K^+^ intake in children with CKD2–5D.
2. To investigate the effectiveness of a Mediterranean or plant-based diet on serum K^+^ levels in children with CKD2–5D.
3. To study the effectiveness of different dietary counseling strategies to lower or increase serum K^+^.
4. To compare the effectiveness and tolerability of dietary interventions versus novel K^+^-binders (zirconium cyclosilicate or patiromer) in controlling serum K^+^.
5. To study the long-term use of novel K^+^-binders (zirconium cyclosilicate or patiromer) to allow a controlled increase in the dietary intake of high nutritional quality K^+^-containing foods. The side effects of these medications require careful study in children with CKD2–5D.
6. Patient and caregiver questionnaires to evaluate burden and quality of life indicators of K^+^-restricted vs. higher K^+^ diets and use of K^+^-binders.

## Supplementary information

ESM 1 (DOCX 430 kb)

## References

[1] Clase CM, Carrero JJ, Ellison DH, Grams ME, Hemmelgarn BR, Jardine MJ, Kovesdy CP, Kline GA, Lindner G, Obrador GT, Palmer BF, Cheung M, Wheeler DC, Winkelmayer WC, Pecoits-Filho R, Conference Participants (2020). Potassium homeostasis and management of dyskalemia in kidney diseases: conclusions from a Kidney Disease: Improving Global Outcomes (KDIGO) Controversies Conference. Kidney Int.

[2] McAlister L, Pugh P, Greenbaum L, Haffner D, Rees L, Anderson C, Desloovere A, Nelms C, Oosterveld M, Paglialonga F, Polderman N, Qizalbash L, Renken-Terhaerdt J, Tuokkola J, Warady B, Van de Walle J, Shaw V, Shroff R (2020). The dietary management of calcium and phosphate in children with CKD stages 2-5 and on dialysis clinical practice recommendation from the Pediatric Renal Nutrition Taskforce (PRNT). Pediatr Nephrol.

[3] Shaw V, Polderman N, Renken-Terhaerdt J, Paglialonga F, Oosterveld M, Tuokkola J, Anderson C, Desloovere A, Greenbaum L, Haffner D, Nelms C, Qizalbash L, Van de Walle J, Warady B, Shroff R, Rees L (2020). Energy and protein requirements for children with CKD stages 2-5 and on dialysis – clinical practice recommendations from the Pediatric Renal Nutrition Taskforce. Pediatr Nephrol.

[4] Guyatt GH, Oxman AD, Kunz R, Atkins D, Brozek J, Vist G, Alderson P, Glasziou P, Falck-Ytter Y, Schunemann HJ (2011). GRADE guidelines: 2. Framing the question and deciding on important outcomes. J Clin Epidemiol.

[5] American Academy of Pediatrics Steering Committee on Quality Improvement and Management (2004). Classifying recommendations for clinical practice guidelines. Pediatrics.

[6] European Food Safety Authority Panel on Dietetic Products, Nutrition and Allergies (NDA) (2016) Dietary reference values for potassium. EFSA J 14:4952

[7] Welch AA, Fransen H, Jenab M, Boutron-Ruault MC, Tumino R, Agnoli C, Ericson U, Johansson I, Ferrari P, Engeset D, Lund E, Lentjes M, Key T, Touvier M, Niravong M, Larrañaga N, Rodríguez L, Ocké MC, Peeters PHM, Tjønneland A, Bjerregaard L, Vasilopoulou E, Dilis V, Linseisen J, Nöthlings U, Riboli E, Slimani N, Binghams S (2009). Variation in intakes of calcium, phosphorus, magnesium, iron and potassium in 10 countries in the European Prospective Investigation into Cancer and Nutrition study. Eur J Clin Nutr.

[8] National Diet and Nutrition Survey: Young people aged 4 to 18 years (2000) Report of the diet and nutrition survey. The Stationery Office, London

[9] National Diet and Nutrition Survey: children aged 1½ to 4½ years (1995) Report of the diet and nutrition survey. The Stationery Office, London

[10] Keast DR, Fulgoni VL, Nicklas AT, O’Neil CE (2013). Food sources of energy and nutrients among children in the United States: National Health and Nutrition Examination Survey 2003–2006. Nutrients.

[11] Cupisti A, Kovesdy CP, D’Alessandro C, Kalantar-Zadeh K (2018). Dietary approach to recurrent or chronic hyperkalaemia in patients with decreased kidney function. Nutrients.

[12] Public Health England (2019) McCance and Widdowson’s ‘Composition of foods integrated dataset’ on the nutrient content of the UK food supply https://www.gov.uk/government/publications/composition-of-foods-integrated-dataset-cofid Accessed 10 July 2020

[13] Regulation (EC) No 1333/2008 of the European Parliament of the Council 16 December 2018 on food additives https://eur-lex.europa.eu/eli/reg/2008/1333/oj Accessed 10 July 2020

[14] Food and Drug Administration (2016) Food labeling: revision of the nutrition and supplement facts labels. Federal Register 81:33894–33895. Accessed 21 May 2020 27236870

[15] Health Canada (2018) The annexed regulations amending the food and drug regulations — nutrition labelling, other labelling provisions and food colours. Accessed 21 May 2020

[16] Sherman RA, Mehta O (2009). Phosphorus and potassium content of enhanced meat and poultry products: Implications for patients who receive dialysis. Clin J Am Soc Nephrol.

[17] Picard K (2019). K additives and bioavailability: are we missing something in hyperkalemia management. J Renal Nutr.

[18] van Buren L, Dötsch-Klerk M, Seewi G, Newson RS (2016). Dietary impact of adding potassium chloride to foods as a sodium reduction technique. Nutrients.

[19] Parpia AS, L’Abbé M, Goldstein M, Arcand J, Magnuson B, Darling PB (2018). The impact of additives on the phosphorus, potassium and sodium content of commonly consumed meat, poultry, and fish products among patients with chronic kidney disease. J Renal Nutr.

[20] Naismith DJ, Braschi A (2008). An investigation into the bioaccessibility of potassium in unprocessed fruits and vegetable. Int J Food Sci Nutr.

[21] Tyson CC, Lin P-H, Corsino L, Batch BC, Allen J, Sapp S, Barnhart H, Nwankwo C, Burroughs J, Svetkey LP (2016). Short-term effects of the DASH diet in adults with moderate chronic kidney disease: a pilot feeding study. Clin Kidney J.

[22] Appel LJ, Moore TJ, Obarzanek E, Vollmer WM, Svetkey LP, Sacks FM, Bray GA, Vogt TM, Cutler JA, Windhauser MM, Lin PH, Karanja N (1997). A clinical trial of the effects of dietary patterns on blood pressure. DASH Collaborative Research Group. N Engl J Med.

[23] Braschi A, Gill L, Naismith DJ (2009). Partial substitution of sodium with potassium in white bread: feasibility and bioavailability. Int J Food Sci Nutr.

[24] Macdonald-Clarke CJ, Martin BR, McCabe LD, McCabe GP, Lachcik PJ, Wastney M, Weaver CM (2016) Bioavailability of potassium from potatoes and potassium gluconate: A randomized dose response trial. Am J Clin Nutr 104:346–353 10.3945/ajcn.115.127225 27413123

[25] Chauveau P, Aparicio M, Bellizzi V, Campbell K, Hong X, Johansson L, Kolko A, Molina P, Sezer S, Wanner C, Ter Wee PM, Teta D, Fouque D, Carrero JJ, European Renal Nutrition (ERN) Working Group of the European Renal Association–European Dialysis Transplant Association (ERA-EDTA) (2018). Mediterranean diet as the diet of choice for patients with chronic kidney disease. Nephrol Dial Transplant.

[26] Kovesdy CP, Appel LJ, Grams ME, Gutekunst L, McCullough PA, Palmer BF, Pitt B, Sica DA, Townsend RR (2017) Potassium homeostasis in health and disease: a scientific workshop cosponsored by the National Kidney Foundation and the American Society of Hypertension. J Am Soc Hypertens 11:783–800 10.1016/j.jash.2017.09.011 29030153

[27] Falkner B (2017). Does potassium deficiency contribute to hypertension in children and adolescents?. Curr Hypertens Rep.

[28] World Health Organization (2012) Guideline: Potassium intake for adults and children. WHO, Geneva, Switzerland 23617019

[29] Biesecker R, Stuart N, Byham-Gray L, Wiesen K (2004). Nutrition management of the adult hemodialysis patient. A clinical guide to nutrition care in kidney disease.

[30] Kopple JD, Shaul GM, Kamyar K-Z (2013) Nutritional management of renal disease, 3rd edn. Elsevier

[31] KDOQI Work Group (2009). KDOQI clinical practice guideline for nutrition in children with CKD: 2008 update. Am J Kidney Dis.

[32] Hui WF, Betoko A, Savant J, Abraham AG, Greenbaum L, Warady B, Moxey-Mims MM, Furth SL (2017). Assessment of dietary intake of children with chronic kidney disease. Pediatr Nephrol.

[33] Chen W, Ducharme-Smith K, Davis L, Wun Fung Hui A, Warady B, Furth SL, Abraham AG, Betoko A (2017). Dietary sources of energy and nutrient intake among children and adolescent with CKD. Pediatr Nephrol.

[34] Tuokkola J, Kiviharju E, Jahnukainen T, Hölttä T (2020). Differences in dietary intake and vitamin and mineral status of infants and children on dialysis receiving feeds or eating normal food. J Renal Nutr.

[35] Hsu CY, Chertow GM (2002) Elevations of serum phosphorus and potassium in mild to moderate chronic renal insufficiency. Nephrol Dial Transplant 17:1419–1425 10.1093/ndt/17.8.1419 12147789

[36] Stover J (2006). Non-dietary causes of hyperkalemia. Nephrol Nurs J.

[37] Zacchia M, Abategiovanni ML, Stratigis S, Capasso G (2016). Potassium: from physiology to clinical implications. Kidney Dis (Basel).

[38] US Department of Agriculture. FoodData Central https://fdc.nal.usda.gov/ Accessed 10 July 2020

[39] Government of Canada. Food and Drug Regulations - division 25 - human milk substitutes and food containing human milk substitutes. https://laws.justice.gc.ca/eng/regulations/C.R.C.,_c._870/page-89.html Accessed 5 May 2020

[40] Bunchman TE (2013). Pretreatment of formula or expressed breast milk with sodium polystyrene sulfonate (Kayexalate®) as a treatment for hyperakalemia in infants with acute of chronic renal insufficiency. J Renal Nutr.

[41] Le Palma K, Pavlick ER, Copelovitch L (2018). Pretreatment of enteral nutrition with sodium polystyrene sulfonate: effective, but beware of the high prevalence of electrolyte derangements in clinical practice. Clin Kidney J.

[42] Thompson K, Flynn J, Okamura D, Zhou L (2013). Pretreatment of formula of expressed breast milk with sodium polystyrene sulfonate (Kayexalate R) as a treatment for hyperkalemia in infants with acute or chronic renal insufficiency. J Renal Nutr.

[43] Cameron JF, Kennedy D, Feber J, Wong E, Geier P, Vaillancourt R (2013). Pretreatment of infant formula with sodium polystyrene sulfonate. Pediatr Drugs.

[44] Rivard AL, Raup SM, Beilman GJ (2004). Sodium polystyrene sulfonate used to reduce the potassium content of a high-protein enteral formula: a quantitative analysis. J Parenter Enter Nutr.

[45] Fassinger N, Dabbagh S, Mukhoppadyay S, Lee D (1998). Mineral content of infant formula after treatment with sodium polystyrene sulfonate or calcium polystyrene sulfonate. Adv Perit Dial.

[46] Picq C, Asplanato M, Bernillon N, Fabre C, Roubeix M, Ricort JM (2014). Effects of water soaking and/or polystyrene sulfonate addition on potassium content of food. Int J Food Sci Nutr.

[47] Schröder CH, van den Berg AMJ, Willems JL, Monnens LAH (1993). Reduction of potassium in drinks by pre-treatment with calcium polystyrene sulphate. Eur J Pediatr.

[48] Taylor JM, Oladitan L, Carslon S, Hamilton-Reeves J (2015). Renal formulas pretreated with medications alters the nutrient profile. Pediatr Nephrol.

[49] Paloian NJ, Bowman B, Bartosh SM (2019). Treatment of infant formula with patiromer dose dependently decreases potassium concentration. Pediatr Nephrol.

[50] Hobbs DJ, Gast TR, Ferguson KB, Bunchman TE, Barletta GM (2010). Nutritional management of hyperkalemic infants with chronic kidney disease, using adult renal formulas. J Ren Nutr.

[51] Alajaji SA, El-Adawy TA (2006). Nutritional composition of chickpea (Cicer arietinum L.) as affected by microwave cooking and other traditional cooking methods. J Food Compos Anal.

[52] Asiimwe J, Sembajwe LF, Senoga A, Bakiika E, Muwonge H, Kalyesubula R (2013). Overnight soaking or boiling of “Matooke” to reduce potassium content for patients with chronic kidney disease: does it really work?. Afr Health Sci.

[53] Bethke PC, Jansky SH (2008). The effects of boiling and leaching on the content of potassium and other minerals in potatoes. J Food Sci.

[54] Burrowes JD, Ramer NJ (2006). Removal of potassium from tuberous root vegetables by leaching. J Renal Nutr.

[55] Burrowes JD, Ramer NJ (2008). Changes in potassium content of different potato varieties after cooking. J Renal Nutr.

[56] Jones LW (2001). Demineralization of a wide variety of foods for the renal patient. J Renal Nutr.

[57] Lima AMS, Dos Santos LO, David JM, Ferreira SLC (2019). Mineral content in mustard leaves according to the cooking method. Food Chem.

[58] Lisiewska Z, Slupski J, Kmiecik W, Gebczynski P (2008). Availability of essential and trace elements in frozen leguminous vegetables prepared for consumption according to the method of pre-freezing processing. Food Chem.

[59] Martínez-Pineda M, Yagüe-Ruiz C, Vercet-Tormo A (2020). Is it possible to include potato in the diet of chronic kidney disease patients? New culinary alternatives for limiting potassium content. J Ren Nutr.

[60] Martínez-Pineda M, Yagüe-Ruiz C, Caverni-Muñoz A, Vercet-Tormo A (2016). Reduction of potassium content of green bean pods and chard by culinary processing. Tools for chronic kidney disease. Nefrologia.

[61] Sousa CT, Soares SAR, Queiroz AFS, dos Santos AMP, Ferreira SLC (2016). Determination and evaluation of the mineral composition of breadfruit (Artocarpus altilis) using multivariate analysis technique. Microchem J.

[62] Wang N, Hatcher DW, Toews R, Gawalko EJ (2009). Influence of cooking and dehulling on nutritional composition of several varieties of lentils (Lens culinaris). LWT Food Sci Technol.

[63] Rondanelli M, Daglia M, Meneghini S, Di Lorenzo A, Peroni G, Faliva MA, Perna S (2017). Nutritional advantages of sous-vide cooking compared to boiling on cereals and legumes: determination of ashes and metals content in ready-to-eat products. Food Sci Nutr.

[64] Goraya N, Simoni JJC, Wesson DE (2013). A comparison of treating metabolic acidosis in CKD stage 4 hypertensive kidney disease with fruit and vegetables or sodium bicarbonate. Clin J Am Soc Nephrol.

[65] Joshi S, Hashmi S, Sanjeev S, Kalantar-Zadeh K (2020). Plant-based diets for prevention and management of chronic kidney disease. Curr Opin Nephrol Hypertens.

[66] Chaitman M, Dixit D, Bridgeman MB (2016). Potassium-binding agents for the clinical management of hyperkalemia. Pharm Ther.

[67] Weir MR, Bakris GL, Bushinsky DA, Mayo MR, Garza D, Stasiv Y, Wittes J, Christ-Schmidt H, Berman L, Pitt B, OPAL-HK Investigators (2015). Patiromer in patients with kidney disease and hyperkalemia receiving RAAS inhibitors. N Engl J Med.

[68] Roger SD, Spinowitz BS, Lerma EV, Singh B, Packham DK, Al-Shurbaji A, Kosiborod M (2019). Efficacy and safety of sodium zirconium cyclosilicate for treatment of hyperkalemia: an 11-month open-label extension of HARMONIZE. Am J Nephrol.

[69] Pitt B, Bakris GL (2015). New potassium binders for the treatment of hyperkalemia: current data and opportunities for the future. Hypertension.

[70] Beccari MV, Meaney CJ (2017). Clinical utility of patiromer, sodium zirconium cyclosilicate, and sodium polystyrene sulfonate for the treatment of hyperkalemia: an evidence-based review. Core Evid.

[71] Kovesdy CP, Rowan CG, Conrad A, Spiegel DM, Fogli J, Nina Oestreicher N, Connaire JJ, Winkelmayer WC (2019). Real-world evaluation of patiromer for the treatment of hyperkalemia in hemodialysis patients. Kidney Int Rep.

[72] Parks M, Grady D (2019). Sodium polystyrene sulfonate for hyperkalemia. JAMA Intern Med.

