# Supplementary material for: The dietary management of potassium in children with CKD stages 2–5 and on dialysis—clinical practice recommendations from the Pediatric Renal Nutrition Taskforce
Source: Pediatr Nephrol. 2021 Mar 17;36(6):1331–46. doi: 10.1007/s00467-021-04923-1 (PMC8084813; doi:10.1007/s00467-021-04923-1)
Supplement: Supplementary file 1 — (DOCX 430 kb) [file 467_2021_4923_MOESM1_ESM.docx]

# The dietary management of potassium in children with CKD stages 2-5 and on dialysis – clinical practice recommendations from the

# Pediatric Renal Nutrition Taskforce

# Supplementary Materials

**Supplementary Table 1: Search terms strategy used in the literature review for dietary management of potassium**

1980 – May 2020 English language

Medline, PubMed, Embase, Cochrane library, Cinahl, manual searching

Given the paucity of studies in this field, all publications, including meta-analyses, randomised controlled trials, prospective studies, retrospective studies (irrespective of patient numbers, including adult and pediatric studies) have been considered.

| 1 | **kidney disease** | **renal failure** | **renal insufficiency** | **chronic kidney disease** | **kidney failure** | **kidney injury** | **kidney dysfunction** | **CKD** |
| --- | --- | --- | --- | --- | --- | --- | --- | --- |
|  | **CRF** | **CKF** | **ESRD** | **ESRF** | **dialysis** | **renal replacement therapy** | **pre dialysis** | **peritoneal dialysis** |
|  | **hemodialysis** | **haemodialysis** | **CAPD** | **APD** |  |  |  |  |
| **2** | **potassium** | **hyperkalemia** | **hypokalemia** |  |  |  |  |  |
| **3** | **diet** | **dietary** | **nutrition** | **food** | **feed** | **intake** | **requirements** |  |
|  | **dietary management** | **dietary advice** | **dietary restriction** | **supplementation** | **dietitian** | **dietician** |  |  |
| **4** | **enteral nutrition** | **tube feeding** | **gastrostomy** | **oral intake** | **potassium intake** |  |  |  |
|  | **nutritional support** | **feeding methods** | **breast feeding** | **human milk** | **infant formula** | **weaning** |  |  |

**Supplementary Table 2:**  **Evidence tables**

| **Potassium intake in children with CKD** | | | | | | |
| --- | --- | --- | --- | --- | --- | --- |
| Study | Population | N | Intervention | Outcome | Remarks | Potential bias/limitations |
| Chen 2017  Cross-sectional study | USA and Canada  age 2-18 yrs  CKD, median (IQR) GFR 53.5 (38.9-73.5)  7.0% patients had hyperkalemia | 658 | FFQ | K intake in 2-3 yr olds:  146 (125, 202) mg/kg/day, AI% 46 ± 15;  4-8 yrs: 108 (79, 135) mg/kg/day, AI% 54 ± 25;  9-13 yrs: 58 (40, 79) mg/kg/day, AI% 53 ± 22;  14-18 yrs: 49 (33, 66) mg/kg/day, AI% 69 ± 31;  Overall: 66 (42, 102)  mg/kg/day  Contribution of dietary sources of K:  Milk 15.9% (vs 18.8% in general population);  fruit excluding bananas (8.5%); fast foods (7.4%); fruit juice (7.0%); potatoes (4.9%); tomato and tomato sauce (3.9%); yogurt (3.4%); banana (3.3%); pork products (3.1%); vegetable and other soup (3.0%); ready-to-eat cereals (3.0%); poultry products (3.0%); milk products (3.0%); carrot, sweet potatoes, squash (2.9%); nuts and seeds (2.3%); other vegetables (2.3%); legumes (2.2%) |  |  |
| Hui 2017  Cross-sectional study | North America  Median age 11 yrs (8-15)  CKD eGFR 30 -90 (using Schwartz formula) or 45-90 (using updated Schwartz formula) | 658 |  | K intake 1-3 years: 2084 mg, 4-8 yrs: 2377 mg, 9-13 yrs: 2334 mg, 14-18 yrs: 2991 mg  Dietary K consumption decreased as CKD advanced  Fruits and vegetables are the main sources of dietary K |  |  |
| Hobbs 2010  Case-series, retrospective report | USA  CKD stages 3-5  Mean age 6.9 mo | 7 | Infants treated with adult renal formulas because of hyperkalemia (initially on Similac PM 40/60 or breastmilk), adult formulas Renalcal/Nestle, Suplena/Ross, Nepro/Ross. Similac mixed with adult renal formlas (and water in polyuric children)  Energy intake 119 vs 121 kcal/kg/day on infant and adult formulas, protein 3.2 g/kg/day on both.  In anuric infants, osmolality of 506+-11 mOsm/kg | K intake decreased from 2.6 to 1 mEq/kg/day, p<0.001, serum K decreased from 5.1 to 4.0 mmol/l, p<0.01  Increase in weight z-score from -1.0 to +0.5, p<0.01, height z-score from -1.9 to -0.5, p<0.01 | Adult renal formulas were well tolerated  Anuric patients tolerated formula well | Small study  no control group |
| Tuokkola 2020  Cross-sectional study | Finland  CKD stage 5D  Median (IQR) age 1.2 (0.8–8.3) yrs | 33 | Food records  21/33 children received renal-specific formula as part of their feed/diet | K intake 455 mg/day: 44 mg/kg/day in anuric patients; 61 mg/kg/day in patients with urine output |  | No control group |

| **Pretreatment of formulas to lower K content** | | | | | | |
| --- | --- | --- | --- | --- | --- | --- |
| Study | Population | N | Intervention | Outcome | Remarks | Potential bias/limitations |
| Bunchman 1991 | USA  3 PD patients  2 chronic renal insufficiency | 5 | Pretreatment of liquids with Na polystyrene sulfonate  Trial in infants, weight 8.6+/-3.2, range 2.8-18kg  Liquids tested: Vanilla forta shake, chocolate milk shake, vanilla instant breakfast/wholemilk, orange juice, chocolate whole milk, Isocal, PM 60/40, citrotein in water, whole milk, apple juice. Dosage: 1 g/1 mEq K | Na polystyrene sulfonate 1g/  1 mEq K  62+/-2.6% (p<0.01) K was removed in 30 min, Na concentration increased by 234 +/- 37% (p<0.01)  Pretreated formula lowered plasma K from 6.94+/-0.15 to 4.10+/-0.12 mEq/l (p<0.01) | Increase in Na | Very small study  No control group  Age of participants not reported |
| Cameron 2013 | Canada | 0 | Pretreatment of infant formula with Na polystyrene  sulfonate in varying concentrations  Dosage: 0.453-2.147 g/mEq K | The addition of 10 ml of SPS resulted in a 53–56% reduction K concentration. With the addition of 20 ml of SPS there were further decreases in K levels (74–77%).  Contact time of 1 or 24 hours did not impact the amount of K removed or the increase in Na concentration | Increase in Na | Not tested if will lower blood concentrations in humans |
| Fassinger 1998 | USA | 0 | Pretreatment of infant formula with Ca polystyrene sulfonate (CPS) and Na polystyrene sulfonate and preparing infant formula with deionized water  Dosage: 1 g/mEq K | CPS decreased K by 12%, SPS by 78%  Preparation with deionized water resulted in 32% and 63% decrease in K and Na concentrations, respectively, when compared to formula made with tap water | CPS reduced Na content, SPS increased Na concentration 3.85 fold  No effect on Zn concentration | Not tested if will lower blood concentrations in humans |
| Le Palma 2018  Retrospective review | USA | 14 | Pretreatment of enteral nutrition with Na polystyrene sulfonate  Serum electrolytes at baseline and after 1 wk of treatment  Formulas: Nutren Jr, Similac Advance, Similac PM 60/40, Peptamen Junior, Renal Cal, Gerber Good Start, Pediasure  Dosage: 0.3–1.0 g/mEq K | Serum K levels decreased from 6.0 to 4.4 mmol/L, serum Na levels increased from 135.8 to 141.3 mmol/L  No differences in S-Ca or S-Mg | Adverse effects: Hypokalemia in 31.6%, hypernatremia in 26.3%, hypocalcemia in 21.1% | Retrospective study |
| Paloian 2019 | USA | 0 | Pretreatment of infant formula with patiromer, a calcium-based cation exchange medication  Dosage: 2.1 g, 8.4 g or  16.8 g per 500 ml | Potassium levels decreased by 20- 54% depending on formula and treatment time with the largest dose Suggested dose 16.8 g/  500 ml with a treatment time of 30 min | Increase in Ca, decrease in Mg, slight decrease in Na, slight increase in P | Not tested if it will lower blood concentrations in humans |
| Rivard 2004 | USA | 0 | Pretreatment of a high-protein enteral formula with SPS  Dosage: 0.5 and 1 g/mEq K | K concentration decreased by 25% and 36% | Na increased by 324% with higher dose of SPS  No change in Mg content, a slight increase in P, Fe Zn  Ca reduced by 14% | Not tested if will lower blood concentrations in humans |
| Schröder 1993 | Netherlands | 0 | Pre-treatment of liquids with Ca polystyrene sulphonate to reduce K content, in 3 concentrations  Liquids tested: infant formula, whole milk, apple juice, orange juice  Dosage: 2, 4, and 8 g per 150 ml of fluid | Dose-response;  with 8 g, average 50% reduction in K | Slightly higher Na contents after treatment  2-9 fold increase in Ca | Not tested if will lower blood concentrations in humans |
| Taylor 2015 | USA | 0 | Pretreatment of Suplena and Similac PM 60/40 with Na polystyrene sulfonate (SPS) suspension and sevelamer carbonate  Dosage: 0.25, 0.5, 1.0 g/mEq K | Pretreatment of Suplena with SPS reduced K concentrations (6–34%)  Pretreatment of Similac PM 60/40 with SPS reduced K concentrations (33–63%)  No differences in K concentration were observed between treatment times  The levels of K were not effectively reduced in Suplena pretreated with sevelamer carbonate alone or when co-administered with SPS | Pretreatment of Suplena with SPS reduced concentrations of:  Ca (11–38 %), Cu (3–11%), Mn (3–16 %), P (0–7%), Zn (5–20 %) and increased those of: Fe (9–34%), Na (89–260%) S (19–45 %) and pH (0.20–0.50 units)  Pretreatment of Similac PM 60/40 with SPS reduced the concentrations of: Ca (8–29%), Cu (5–19%), Mg (3–26%) and increased  those of: Fe (13–87%) and Na (86–247%) and pH (0.40–0.81 units)  Pretreatment of both formulas with the SPS  suspension led to significant increases in the Al concentration in both formulas (507–3957%) | Not tested if will lower blood concentrations in humans |
| Thompson 2013  Retrospective | USA  Infants with CKD or AKI with hyperkalemia, GFR 4-62  Age 1.4-33 weeks | 13 | Pretreatment of formula (Similac PM 60/40) or expressed breastmilk with  Na polystyrene  Dosage: 0.9 (0.4-1.5) g/100 ml feed | 24% reduction in serum K after consumption of pretreated formula (P < 0.0001)  Decrease in serum Ca and creatinine levels (P<0.05), no difference in other electrolytes (2% increase in Na, NS)  Measurements of K in formula/EBM: 18% and 21% reduction of K in 0.25 and 0.5 g batches (P <0.001) and a 50% reduction of K content in the 1.0 g/100 ml batch (P<0.0001) compared with 2 control samples | No adverse effects reported | Retrospective study |

| **Demineralization of foods** | | | | | | |
| --- | --- | --- | --- | --- | --- | --- |
| Study | Population | N | Intervention | Outcome | Remarks | Potential bias/limitations |
| Alajaji 2006 | Saudi Arabia  Chick peas | 0 | Soaking 12 h, boiling/ autoclaving/microwave cooking until soft, amount of water 1:10 in all | Reduction in K:  Boiling 61%, autoclaving 53%, microwave cooking 50% | Reductions by all methods also in rIboflavin, thiamin, niacin, pyridoxine, Ca, Mn, Zn, Cu, Fe, P | Effects on human blood K concentrations not studied  Object of the study was not to reduce K |
| Asiimwe 2013 | Uganda  Matooke bananas | 0 | Peeled; soaked for 2-24 h, boiled at 200⁰ C for 10-60 min | Soaking: no effect on K content  Boiling, K reduction 35% |  | Effects on human blood K concentrations not studied |
| Bethke 2008 | USA  Potatoes, 6 varieties | 0 | Peeled, diced 1 cm cubes /shredded  Soaking (30 g/300 ml) 20 h at 5.6⁰ C  + boiling in 300 ml, 10 min | Differences in K content between varieties  Leaching: 0-4% reduction in K  Boiling diced: 50% reduction  Boiling shredded: 69-75% reduction | Other minerals: P, Mg, S, Zn, Mn and Fe levels were significantly reduced following leaching plus boiling or boiling alone treatments  Ca, B and Cu levels did not always follow this trend  Mg, S, Mn and Zn were all reduced  by an average of 50% or more in the shredding and boiling  treatment | Effects on human blood K concentrations not studied |
| Burrowes 2006 | USA  Tuberous root vegetables: sweet potato, cocomalanga, dasheen, eddo, yam (black, white, yellow), yampi, malanga, yautia (red, white), cassava | 0 | Sliced to 3 mm, cooked 5-10 min, 2:1 water-to-sample ratio  No soak + cooking  2 h soak + cooking  4 h soak + cooking  8 h soak + cooking  No soak + double cooking | Soaking the vegetables for 2 hours, 4 hours and 8 hours before normal cooking was not effective in leaching significant amounts of  K from most of the vegetables  Reduction of 22-66% K by cooking, 40-68% by double-cooking |  | Effects on human blood K concentrations not studied |
| Burrowes 2008 | USA  6 different potato varieties | 0 | Normal cooking: water at 2:1 water-to-sample ratio, cooking 5-10 min  double cooking: water first brought to boil, then changed and boiled again  Peeled, sliced to 3 mm | In raw potatoes, K content 295-448 mg/100g  Reduction in K from normal to double cooking 3-38% in different varieties  Reduction in K from raw to double cooking 39-60% in different varieties |  | Effects on human blood K concentrations not studied |
| Jones 2001 | USA  Several vegetables, legumes, meats, flours, cheddar cheese and fruit | 0 | Foods cut into ¼ inch slices  Processed in water in different temperatures (hot tap water/boiling water/cold water) and different times  After that soaked in 2 L of water | Reduction range of K: vegetables 59% ± 40%, legumes 78.5% ±20.5%, meats 57% ± 41%, flours 94% ± 3%, cheddar cheese 99%, fruit 43%±16% | Texture and visual appeal considered satisfactory | Effects on human blood K concentrations not studied  Food processing was variable |
| Lima 2019 | Brazil  Mustard leaves | 0 | Cooked (2 g/50 ml) on the stove 20 min, in a microwave oven 5 min,  water bath 20 min | Reduction in K: water bath 16%, stove 53%, microwave 77% | Reductions also in Ba, Ca, Fe, Mg, Na, P, S, Zn | Effects on human blood K concentrations not studied |
| Lisiewska 2008 | Poland  Pea, broad bean, French bean | 0 | 2 methods: 1) blanching, freezing and cooking; 2) cooking, freezing and defrosting and heating in microwave oven | Reduction in K:  broad bean: 1) 26%; 2) 14%  pea: 1) 39%; 2) 26%  French bean: 41%; 2) 20% | Reductions also in P, Mg, Fe, Zn, Mn, Cu, Cr | Effects on human blood K concentrations not studied  Object of the study was not to reduce K |
| Martinez-Pineda 2019 | Spain  Fresh potatoes (S. tuberosum cv. Kennebec), canned potatoes (three brands), frozen french fried potatoes (four brands)  Strips and diced | 0 | Soaking, normal cooking, frying  Fresh: strips 1.2 x 1.2 cm and diced 2 x 2 x 2 cm  French fries: diced (2.5 x 2.3 x 2.8 cm), strips (9 x 9 mm) and extrafine strips  (6 x 6 mm)  Soaking 12 h, with and without changing water after 4 h  Cooking 8 min, water 100 g/1.5 L, 200 g/1.5 L and 300 g/1.5 L for potatoes cut  into strips; and 100 g/1.5 L for diced potatoes | Soaking ineffective for fresh raw potato  Boiling removed 30%  soaking after boiling: 70%-85% of K leached (diced, strip-cut)  Frying increased K content  Strips vs. cubes: no difference  Most K removed with 6 h soaking, extended to 12 h removed only 5-10% more  French fries: soaking with changed water removed 10-38% K  Soaked potatoes: K content low in the beginning, leaching removed 70% | Final K content fresh potatoes: 40-120 mg/100 g edible portion  Canned potatoes: 35 mg/100 g  Sensory analysis and texture: good marks | Effects on human blood K concentrations not studied |
| Martinez-Pineda 2018 | Spain  Chickpeas, lentils, both dried and canned | 0 | Soaking at different pH for 12 h, 100 g of product/1.5 L water  Normal cooking  Pressure cooking | Minor loss by soaking, not clinically relevant  After soaking  and cooking dried chickpeas, the retention percentage of  K was 15-30%  Lentils: normal and pressure cooking reduced K to 27% and 37% of original  Canned chickpeas: normal cooking, 35%, soaking in water 18%, soaking in water + normal cooking 6.4%; Lentils normal cooking 39%, soaking in water 18%, soaking in water + normal cooking 5.6% from the original |  | Effects on human blood K concentrations not studied |
| Martinez-Pineda 2016 | Spain  Green beans and chard; fresh, frozen, canned  4 different brands of frozen mixed vegetables  Diced vegetable salad | 0 | Soaking (12 h in refrigeration temperature, change of water at 4 h); soaking followed by normal cooking (S + NC); soaking followed by  double cooking (S + DC); normal cooking (NC); double cooking  (DC)  No soaking or cooking of canned products | Reduction % K:  Green beans: soaking 15% (fresh); 93% (frozen); normal cooking 33% (fresh) - 64% (frozen); double cooking 33% (fresh) -75% (frozen)  Chard: soaking 20% (fresh); 90-98% (frozen); normal cooking 47% (fresh) – 76% (frozen); double cooking 66% (fresh) - 90% (frozen)  Mixed vegetables: cooking, 22-50% reduction |  | Effects on human blood K concentrations not studied |
| Picq 2014 | France  Chocolate, apple, banana,  tomato and potato | 0 | Na polystyrene sulfonate added to liquids (2, 4, or 6 g/100 ml)  Solid foods peeled, sliced, 100 g soaked in 1 L of water with or without SPS (6 or 12 g)  Potatoes peeled, sliced, soaked, boiled with SPS (6, 12, 48 g/L) | Reduction of K by 53% and 73% in milk and by 43% and 74% in orange juice with SPS 2% and 4–6%  Water soaking reduced  K content (by 16% for chocolate and potato, 26% for apple, 37% for tomato and 41% for banana)  Addition of Na polystyrene sulfonate to the soaking water did not further increase K loss from the five foods tested  Boiling reduced K content of potatoes by 73%, SPS had no additive effect | Na polystyrene sulfonate resin treatment induced a significant  dose-dependent increase in Na content that reached similar values in semi-skimmed milk and orange juice whatever the initial Na concentration | Effects on human blood K concentrations not studied |
| Rondanelli 2016 | Italy  Red lentils, peas, borlotti beans, pearl barley, cereals soup | 0 | Normal and sous-vide cooking | K content higher after sous-vide cooking than normal cooking |  | Effects on human blood K concentrations not studied  Study not intended to reduce K |
| Sousa 2016 | Brazil  Breadfruit | 0 | Boiling 5 min or microwave heating 4 min, 1g/25 ml water | Reduction in K: boiling 48%, microwave 32% | Reductions also in Ca, P, Mg, Fe, Na, Mn | Effects on human blood K concentrations not studied |
| Wang 2009 | Canada  lentils | 0 | Soaking in water 24 h (1:4), then cooking (100 g in 2L water until soft) or dehulling (3 days) | Cooking: reduction in K  Dehulling: increase in K | Cooking lentils in boiling water resulted in a significant (p <0.05) increase in Ca, Cu, Mn and losses of Fe, Mg, P, Zn  Dehulling significantly  (p <0.05) decreased Ca, Cu, Fe, Mg, Mn and increased P | Effects on human blood K concentrations not studied  Object of the study was not to reduce K |

| **Food additives** | | | | | | |
| --- | --- | --- | --- | --- | --- | --- |
| Study | Population | N | Intervention | Outcome | Remarks | Potential bias/limitations |
| Parpia 2018  Cross sectional study | Meat and poultry products (MPPs) | 0 | The protein, Na, P and K  contents of Na-reduced MPPs and the non-Na-reduced (original) MPP  counterparts were chemically analyzed | Na-reduced MPPs contained 44% more K (mg/100 g) than  their non-Na-reduced counterparts (mean difference [95% CI): 184 [90-  279]; p=0.001)  The K content of Na-reduced MPPs varied widely and  ranged from 210 to 1,500 mg/100 g  K-containing additives were found on the ingredient list in 63% of the Na-reduced products and 26% of the non-Na-reduced products (p=0.02) |  | Analysis of MPPs is from one geographical area Formulations of the products can change over time, thus altering the nutritional content |

| **Plant based diets/potassium-fiber ratio** | | | | | | |
| --- | --- | --- | --- | --- | --- | --- |
| Study | Population | N | Intervention | Outcome | Remarks | Potential bias/limitations |
| Goraya 2013  Randomised study | Adults; stage 4 with metabolic acidosis | 76 | N=35 oral NaHC03; N=37 base-producing fruits and vegetables; period of one year | eGFR did not differ at baseline and 1 year  One-year plasma total CO_2_  was higher than baseline in the HCO_3_ group (21.2+1.3 versus 19.5+1.5 mM; p<0.01) and the fruits and vegetables  group (19.9+1.7 versus 19.3+1.9 mM; p<0.01), consistent with improved metabolic acidosis and was  higher in the HCO_3_ than the fruits and vegetable group (p<0.001)  One-year urine indices of kidney injury were lower than baseline in both groups Plasma K did not increase in either group | Study criteria probably excluded  individuals at high risk for hyperkalemia | Aim of this study was not to research the results of fruits and vegetables on serum K |

| **Bioavailability** | | | | | | |
| --- | --- | --- | --- | --- | --- | --- |
| Study | Population | N | Intervention | Outcome | Remarks | Potential bias/limitations |
| Naismith 2008  Cross-over study | Students/ adults | 11 | 10 day cross-over feeding trial; 5 days with high bio-available K and 5 days low bio-available K | K bioavailability of fruit and vegetables = 76.8%  K bioavailability of fruit juice and meat = 96.3% | Lower bioavailability of fruit and vegetables is attributed to the cellular structure of plant foods |  |
| Tyson 2016  Prospective  study | Adults eGFR 30-59 ml/min/1.73m^2^; hypertension with medication | 11 | A prospective before–after feeding study providing a reduced-Na, run-in diet for 1 week followed by a reduced Na, DASH diet for 2 weeks | DASH modestly increased serum K at 1 week (mean ± SD, +0.28 ± 0.4 mg/dL; p = 0.043) no significant effect on K at 2 weeks (+0.15 ± 0.28 mg/dL; p = 0.13)  Serum bicarbonate was reduced (−2.5 ± 3.0 mg/dL; p = 0.03) at 2 weeks  Neither incidence of hyperkalemia nor new onset metabolic acidosis was observed  Clinic BP and mean 24-h  ambulatory BP was unchanged  DASH significantly reduced mean night-time BP (−5.3 ± 5.8 mmHg; p = 0.018) |  | Small sample size, which limits power; lack of a control group; short duration of feeding |
| Appel 1997 | Adults with systolic blood  pressures of less than 160 mm Hg and diastolic  blood pressures of 80 to 95 mm Hg | 459 | For 3 weeks, a control diet low in fruits, vegetables and dairy products, with a fat content typical of the average diet in the United States  Then randomly assigned to receive for 8 weeks a diet, rich in fruits and vegetables, or a “combination” diet rich in fruits, vegetables and low-fat dairy products and with reduced saturated and total fat | At base line, the mean (+SD) systolic 131.3+10.8 mm Hg and diastolic BP 84.7+4.7 mm Hg  The combination  diet reduced systolic and diastolic BP by 5.5 and 3.0 mm Hg more, respectively, than the control diet (p<0.001 for each); the fruits-and-vegetables diet reduced systolic BP by 2.8 mm Hg  more (p<0.001) and diastolic BP by 1.1 mm Hg more (p<0.07) than the control diet  Among the 133 subjects with hypertension (systolic pressure > 140 mm Hg; diastolic pressure >90 mm Hg; or both), the combination diet reduced systolic and  diastolic BP by 11.4 and 5.5 mm Hg more, respectively, than the control diet (p<0.001 for each); among the 326 subjects without hypertension, the corresponding reductions were 3.5 mm Hg (p<0.001) and 2.1 mm Hg (p<0.003) |  |  |
| Macdonald-Clarke 2016  Single-blind cross-over  randomized controlled trial; dose response trial | Adults | 35 | For nine 5-d interventions of additional K as follows: 0 (control; repeated at phases 1 and 5), 20, 40 and 60 mEq K/d consumed as a K gluconate supplement or  as unfried potato or 40 mEq K from French fries completed at phase 9  The bioavailability of K was determined from AUC of serial blood draws and cumulative urinary  excretion during a 24-h period and from a kinetic analysis  The effects of the K source and dose on the change in BP and AIx were determined | The serum K AUC increased with the dose (p <0.0001) and did not differ due to the source (p = 0.53)  Cumulative 24-h urinary K also increased with the dose (p = 0.0001) and was greater with potato than with the supplement (p = 0.0001)  The kinetic analysis showed the absorption efficiency was high across all interventions (>94% + 12%)  There were no significant differences in the change in blood pressure or AIx with the treatment source or dose | The results suggest that the bioavailability of K is higher from potatoes than from supplements, which is a positive message for using food when a higher K intake is required | Small study; in normotensive population, short duration; no balance study |
| Braschi 2009  Single-blind cross-over feeding trial | Adults | 41 | Two breads in which 30% of Na was replaced by K salts, and bread  in which 10% of wheat flour was replaced with soy flour | The mean bioavailability of the supplementary K  (22.0 mmol/day) was found to be 113.2 +/- 11.20% |  |  |

**Supplementary Table 3: American Academy of Pediatrics grading matrix**


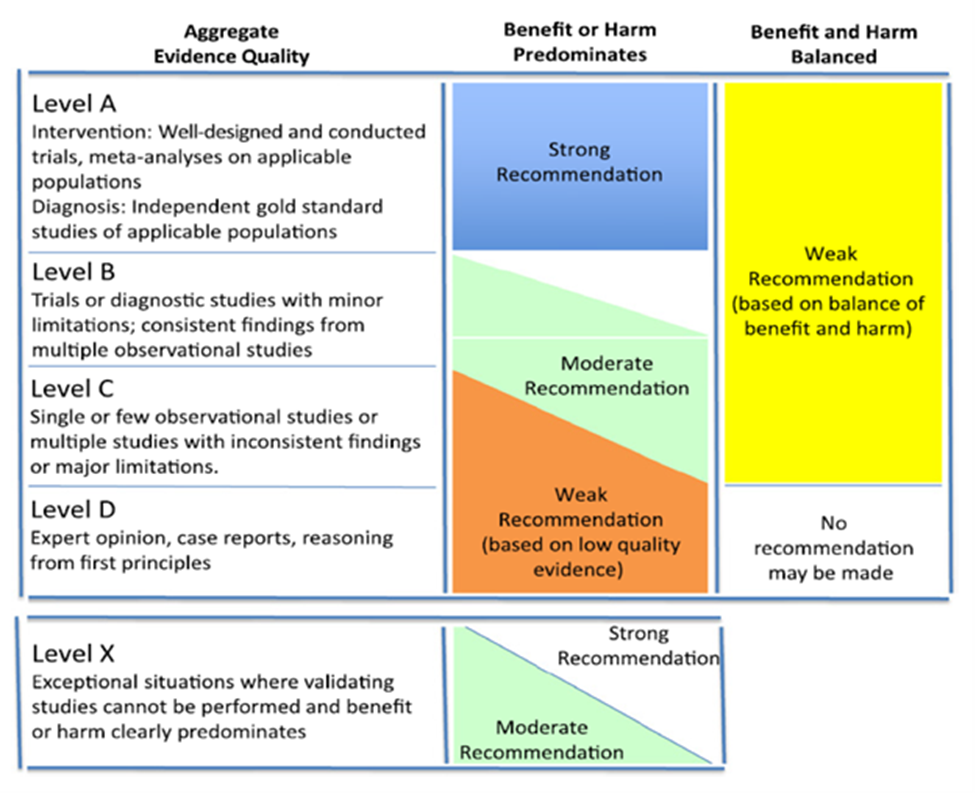


**Supplementary Table 4a: Percentage contribution of food types to average daily potassium (K) intake by age and sex (8, 9)**

Data adapted from UK National Diet and Nutrition Survey (1995-2000), for children aged from 18 months to 18 years

| % total dietary K intake | | | | | | | |
| --- | --- | --- | --- | --- | --- | --- | --- |
| Food group | Age (years) | | | | | | |
|  | 1.5-2.5 | 2.5-3.5 | 3.5-4.5 (b, g) | 4-6 (b, g) | 7-10 (b, g) | 11-14 (b, g) | 15-18 (b, g) |
| Cereals & cereal products | 12 | 14 | 16, 15 | 15, 14 | 17, 15 | 16, 14 | 15, 14 |
| Milk & milk products | 37 | 30 | 26, 26 | 24, 24 | 20, 17 | 18, 14 | 14, 13 |
| Eggs & egg dishes | 1 | 1 | 1, 1 | 1, 1 | 1, 1 | 1,1 | 1,1 |
| Fat spreads | 0 | 0 | 0 | 0 | 0 | 0 | 0 |
| Meat & meat products | 7 | 8 | 9, 9 | 10, 10 | 11, 12 | 14, 13 | 16, 13 |
| Fish & fish dishes | 2 | 2 | 2, 2 | 2, 2 | 2, 2 | 2, 2 | 2,2 |
| Vegetables, potatoes & savoury snacks | 21 | 24 | 25, 27 | 31, 30 | 32, 35 | 35, 40 | 36, 38 |
| Fruit & nuts | 8 | 8 | 8, 7 | 7, 8 | 6, 7 | 4, 4 | 3, 5 |
| Sugars, preserves & confectionery | 2 | 3 | 3, 3 | 2, 3 | 3, 3 | 3, 3 | 3, 3 |
| Beverages/drinks* | 8 | 8 | 8, 7 | 5, 6 | 6, 6 | 6, 6 | 9, 9 |
| of which fruit juice | - | - | - | 3, 4 | 3, 4 | 3, 4 | 3, 4 |
| Commercial infant foods & drinks | 1 | 0 | 0, 0 | - | - | - | - |
| Miscellaneous** | 2 | 2 | 2, 2 | 2, 2 | 2, 2 | 2, 2 | 2, 3 |
| Average daily intake (mg) | 1476 | 1513 | 1573, 1501 | 1944, 1774 | 2136, 2019 | 2392, 2100 | 2833, 2162 |
| Total number of children/young people | 576 | 606 | 250, 243 | 184, 171 | 256, 226 | 237, 238 | 179, 210 |

b, boy; g, girl

*NDNS 4-18 years includes soft drinks, alcoholic drinks, tea, coffee and water

** NDNS 4-18 years includes powdered beverages (except tea and coffee), soups, sauces, condiments and commercial toddlers’ foods

**Supplementary Table 4b: Minimum and maximum percentage contribution of food types to daily potassium (K) intake by age and sex (6)**

Data adapted from European Food Safety Authority Panel on Dietetic Products (2016), for children aged 1-18 years

| % total dietary K intake (minimum – maximum) | | | | |
| --- | --- | --- | --- | --- |
| Food group | Age (year) | | | |
|  | < 1 (b;g) | 1 to < 3 (b;g) | 3 to < 10 (b;g) | 10 to < 18 (b;g) |
| Additives, flavours, baking and processing aids | < 1 | < 1, 0 | 0 | < 1-1 |
| Alcoholic beverages | < 1 | < 1 | < 1 | < 1-1, < 1 |
| Animal and vegetable fats and oils | < 1 | < 1 | < 1 | < 1 |
| Coffee, cocoa, tea and infusions | < 1-2; < 1-15* | < 1-5 | 1-8 | 2-6 |
| Composite dishes | < 1-4; < 1-2 | < 1-7 | < 1-7 | < 1-10 |
| Eggs and egg products | < 1 | < 1-1 | < 1-1 | < 1-1 |
| Fish, seafood, amphibians, reptiles and invertebrates | < 1-1; 0 | < 1-4; < 1-6 | < 1-4; < 1-3 | < 1-4 |
| Food products for young people | 20-54; 19-57 | 3-16 | < 1-1 | < 1 |
| Fruit and fruit products | 5-14; 8-12 | 9-14 | 6-11; 7-12 | 4-9; 6-15 |
| Fruit and vegetable juices an nectars | < 1-2 | 1-8; 1-7 | 4-10; 3-10 | 4-10; 3-10 |
| Grains and grain-based products | 3-6; 4-6 | 8-14; 9-14 | 9-19; 9-18 | 11-20; 12-19 |
| Human milk | < 1-26; < 1-9 | < 1-1 | - | - |
| Legumes, nuts, oilseeds and spices | < 1-2 | 1-3 | 1-4 | 1-4 |
| Meat and meat products | < 1-4 | 4-8; 4-7 | 6-13; 5-14 | 8-16; 7-14 |
| Milk and dairy products | 7-18; 4-22 | 25-34; 23-38 | 17-36; 17-37 | 13-30; 11-27 |
| Products for non-standard diets, food imitates and food supplements or fortifying agents | < 1 | 0; < 1 | < 1-1; 0-1 | < 1-1 |
| Seasoning, sauces an condiments | < 1-1 | < 1-1 | < 1-2 | < 1-2 |
| Starchy roots or tubers and products thereof, sugar plants | 1-21; 4-20 | 6-19; 6-17 | 10-18; 10-19 | 12-21; 11-23 |
| Sugar, confectionery and water-based sweet desserts | < 1; < 1-1 | < 1-1 | < 1-2; 1-3 | < 1-2 |
| Vegetables and vegetable products | 1-15; 4-17 | 5-10; 6-12 | 7-16; 8-16 | 7-19; 8-20 |
| Water and water-based beverages | < 1; < 1-1 | < 1-1 | < 1-2; < 1-3 | < 1-2 |

b, boy; g, girl; when one figure only is given, there is no difference in intake between sexes.

*The value of 15% comes from the INRAN_SCAI_2005_06 survey (girls <1 year n = 7) and originates from one subject who drank small amounts of tea on each of the three days of the survey.

**Supplementary Table 4c: Percentage contribution of food types to mean daily potassium (K) intake by age (10)**

Data adapted from US National Health and Nutrition Examination Survey (2003-2006), for children aged 2-18 years

| Mean K intake (%) | | | |
| --- | --- | --- | --- |
| Food group | Age (years) | | |
|  | 2-5 | 6-11 | 12-18 |
| Milk | 21.1 | 15.6 | 15.0 |
| Fruits | 9.6 | 7.3 | 5.6 |
| 100% juice | 8.5 | 5.7 | 4.6 |
| Flavored milk | 5.1 | 5.3 | 2.5 |
| Mixed dishes – grain based | 3.7 | 4.5 | 3.7 |
| Vegetables, excluding potatoes | 3.9 | 3.7 | 4.1 |
| White potatoes | 3.9 | 3.7 | 6.1 |
| Poultry | 3.5 | 3.6 | 4.5 |
| Savory snacks | 3.1 | 4.0 | 4.0 |
| Sweetened beverages | 3.0 | 3.9 | 2.8 |
| Cured meats/ poultry | 3.0 | 3.4 | 3.1 |
| Yoghurt | 2.8 | 3.4 | - |
| Breads, rolls, tortillas | 2.5 | 2.9 | 2.7 |
| Planted-based protein foods | 2.4 | 2.9 | 2.6 |
| Mixed dishes – Mexican | 1.8 | 2.8 | 3.9 |
| Sweet bakery products | 1.9 | 2.7 | 2.0 |
| Mixed dishes – pizza | 1.6 | 1.6 | 3.6 |
| Ready to eat cereals | 1.9 | 2.4 | 1.7 |
| Mixed dishes – meat/poultry/fish | 1.6 | 2.4 | 2.9 |
| Other desserts | 1.0 | 2.1 | 1.1 |
| Mixed dishes – sandwiches | 1.4 | 1.7 | 3.1 |
| Mixed dishes – soup | 1.5 | 1.7 | 1.3 |
| Eggs | 1.0 | 1.4 | 0.9 |
| Meats | 1.2 | 1.6 | 3.7 |
| Quick bread and bread products | 0.8 | 0.9 | - |
| Mixed dishes – Asian | - | - | 2.0 |
| Coffee and tea | - | - | 1.9 |
| Condiments and sauces | - | - | 2.0 |

The table contains the adjusted intake, i.e. the total daily intake after nutrients from dairy and from non-dairy food (e.g. mixed dishes) has been included. This concerns food sources that contributed at least 1% of potassium intake before adjusting.

**Supplementary Table 5: Approved additives and E-numbers containing potassium**

| *Preservatives* |  |
| --- | --- |
| E202 | Potassium sorbate |
| E212 | Potassium benzoate |
| E224 | Potassium metabisulphite |
| E228 | Potassium hydrogen sulphite |
| E249 | Potassium nitrite |
| E252 | Potassium nitrate |
| E283 | Potassium propionate |
| *Sweeteners* |  |
| E950 | Acesulfame K |
| E954 | Saccharin and its Na, K and Ca salts |
| *Emulsifiers, stabilisers, thickeners and gelling agents* |  |
| E402 | Potassium alginate |
| E470a | Sodium, potassium and calcium salts of fatty acids |
| *Others** |  |
| E261 | Potassium acetate |
| E326 | Potassium lactate |
| E332 | Potassium citrates |
| E336 | Potassium tartrates |
| E337 | Sodium potassium tartrate |
| E340 | Potassium phosphates |
| E351 | Potassium malate |
| E357 | Potassium adipate |
| E501 | Potassium carbonates |
| E508 | Potassium chloride |
| E515 | Potassium sulphates |
| E522 | Aluminium potassium sulphate |
| E525 | Potassium hydroxide |
| E536 | Potassium ferrocyanide |
| E555 | Potassium aluminium silicate |
| E577 | Potassium gluconate |
| E622 | Monopotassium glutamate |
| E628 | Dipotassium guanylate |
| E632 | Dipotassium inosinate |

*Acid, acidity regulators, anti-caking agents, anti-foaming agents, bulking agents, carriers and carrier solvents, emulsifying salts, firming agents, flavour enhancers, flour treatment agents, foaming agents, glazing agents, humectants, modified starches, packaging gases, propellants, raising agents and sequestrants.

**Supplementary Table 6: Potassium content of medicines**

Potassium in medicines can appear in three ways:

- As part of the active ingredient.
- A small number of medicines are formulated as the potassium salt or contain potassium as an active ingredient.
- Other ingredients used to formulate medicines may contain potassium. This is more difficult to track and may not always be obvious from the ingredient list. The amount is presumed to be small and is not generally an issue.

Examples of medicines containing potassium salts:

- penicillins with clavulanic acid (e.g. Co-amoxiclav®)
- angiotensin receptor blockers (ARB) (e.g. Losartan®)
- rehydration salts (e.g. Dioralyte® oral rehydration solution)
- antacids (e.g. Gaviscon®)

The table below shows medicines where the potassium content is known.

| **Drug** | **Formulation*** | **Potassium (mg)** | **Potassium (mmol)** |
| --- | --- | --- | --- |
| Co-amoxiclav® | 125/31.25mg/5ml oral suspension | 5.95 | 0.15 |
| Co-amoxiclav® | 250/62.5mg/5ml oral suspension | 11.9 | 0.31 |
| CoasmolCol® | 6.9g powder for oral solution | 11.7 | 0.30 |
| CoasmolCol®** | 13.1g powder for oral solution | 23.4 | 0.60 |
| Losartan® | 12.5mg tablets | 1.06 | 0.03 |
| Losartan® | 25mg tablets | 2.12 | 0.05 |
| Losartan® | 50mg tablets | 4.24 | 0.11 |
| Losartan® | 100mg tablets | 8.48 | 0.22 |
| Dioralyte® | Sachet | 157.9 | 4.05 |
| Sandoz oral rehydration solution® | Sachet | 157.1 | 4.02 |
| Gaviscon® | Oral suspension (per 5ml) | 39 | 1.00 |
| Phenoxymethyl Penicillin ® | 125mg/5ml oral solution | 12.5 | 0.32 |
| Phenoxymethyl Penicillin® | 250mg/5ml oral solution | 25 | 0.64 |

 Source: Farmacotherapeutisch Kompas <https://www.farmacotherapeutischkompas.nl>

*Potassium content may vary depending on different formulations/manufacturers.

** Also available as Movicol® 13.7g sachet with an equivalent potassium content.

Hypokalemia can result from the use of medicines as thiazide diuretics, loop diuretics, laxatives, corticosteroids, potassium-sparing diuretics, mainly in combination with angiotensin converting enzyme (ACE) inhibitors and ARBs.

**Supplementary Table 7: International recommendations for potassium in healthy children**

| **DRV Potassium (mg/day)** | | | | | |
| --- | --- | --- | --- | --- | --- |
| Age | NA (2019) ^(a)^ | DACH (2016) ^(b)^ | EFSA (2016) ^(a)^ | NCM (2012) ^(c)^ | NHMRC (2006) ^( a)^ |
| Months | 0-6  400 | 0-4  400 |  |  | 0-6  400 |
|  | 7-12  860 | 4<12  600 | 7-12  700 | 6-11  1100 | 7-12  700 |
|  |  |  |  | 12-23  1400 |  |
| Years | 1-3  2000 | 1<4  1100 | 1-3  800 | 2-5  1800 | 1-3  2000 |
|  | 4-8  2300 | 4<7  1300 | 4-6  1100 | 6-9  2000 | 4-8  2300 |
|  | 9-13  2500/ 2300 * | 7<10  2000 | 7-10  1800 | 10-13  3300/2900 * | 9-13  3000/2500 * |
|  | 14-18  3000/ 2300 * | 10<13  2900 | 11-14  2700 | >14  3500/3100 * | 14-18  3600/2600 * |
|  |  | 13<15  3600 | >15  3500 |  |  |
|  |  | >15  4000 |  |  |  |

DRV Dietary Reference Value.

(a) Adequate Intake; (b) Adequate Minimum Intake; (c) Population Reference Intake

*boys/girls

**Abbreviations for health organizations and their rationale for recommendations:**

NA: National Academies of Sciences US and Canada; for infants based on the intake of breastmilk; from 6 months breastmilk and complementary foods; from 1-18 years based on food surveys from USA (NHANES 2009-2014) and Canada (CCHS 2017).

DACH: Deutschland-Austria-Confoederatio Helvetica; based on the needs to maintain electrolyte homeostasis and for cellular growth.

EFSA: European Food Safety Authority; extrapolated from adult values based on body weights and a growth factor.

NCM: Nordic Council of Ministers (also followed by The Netherlands); extrapolated from adult values based on difference in body weight and needs for growth.

NHMRC: National Health and Medical Research Council (Australia and New Zealand); for infants based on the intake of breastmilk; from 6 months breastmilk and complementary foods, using data from US (IOM 2004); from 1-18 years based on food surveys from Australia (ABS 1998) and New Zealand (MOH 2003).

Scientific Committee for Food Luxembourg (SCF 1993) and Department of Health UK (DH 1991) have not been included as the data are not considered current.

World Health Organization (WHO 2012): *The recommended potassium intake of at least 90 mmol/day (3500 mg/day) for adults should be adjusted downwards for children, based on the energy requirements of children relative to those of adults.* These data are not included as the suggested extrapolation is not clear.

**References:**

Australian Bureau of Statistics: Department of Health and Aged Care; National nutrition survey. Nutrient intakes and physical measurements. Australia, 1995. Canberra: Australian Bureau of Statistics, 1998.

Canadian Community Health Survey Nutrition (CCHS-Nutrition). 2017 Reference Guide to Understanding and Using the Data 2015 Canadian Health Survey. <https://www.canada.ca/en/health-canada/services/food-nutrition/food-nutrition-surveillance/health-nutrition-surveys/canadian-community-health-survey-cchs/reference-guide-understanding-using-data-2015.html>

Department of Health. Dietary reference values for food energy and nutrients for the United Kingdom. Report of the Panel on Dietary Reference Values of the Committee on Medical Aspects of Food Policy. HMSO, London, UK, 1991.

Deutsche Gesellschaft für Ernährung, Österreichische Gesellschaft für Ernährung, Schweizerische Gesellschaft für Ernährung, 2015. Referenzwerte für die Nährstoffzufuhr. 2. Auflage, 1. Ausgabe. DGE, Bonn, Germany.

Food and Nutrition Board: Institute of Medicine. Dietary Reference Intakes for water, potassium, sodium, chloride and sulfate. Panel on the dietary reference intakes for electrolytes and water. Washington, DC: National Academy Press, 2004.

 Institute of Medicine. Dietary reference intakes for water, K, sodium, chloride and sulfate.  Washington, DC: The National Academies Press (US), 2005.

Ministry of Health. [NZ](https://www.nrv.gov.au/glossary#NZ) Food [NZ](https://www.nrv.gov.au/glossary#NZ) Children. Key results of the 2002 National Children's Nutrition Survey. Wellington: Ministry of Health, 2003.

National Academies of Sciences, Engineering, and Medicine. Dietary Reference Intakes for sodium and potassium. Washington, DC: The National Academies Press, 2019.

National Health and Medical Research Council, Australian Government Department of Health and Ageing, New Zealand Ministry of Health. Nutrient Reference Values for Australia and New Zealand. Canberra: National Health and Medical Research Council, 2006.

 Nordic Council of Medicine. Nordic Nutrition Recommendations 2012 5th edition – Integrating nutrition and physical activity, 2012.

Scientific Committee for Food. Nutrient and energy intakes for the European Community. Reports of the Scientific Committee for Food, 31st Series. Food- Science and Technique, European Commission, Luxembourg, 1993.

Strohm D. Revised Reference Values for Potassium Intake. Ann Nutr Metab 2017;71:118-124 DOI: 10.1159/000479705

World Health Organization. Diet, nutrition and the prevention of chronic disease. Report of a joint WHO/FAO expert consultation. Geneva, WHO, 2003.

World Health Organization. Effect of increased potassium intake on blood pressure, renal function, blood lipids and other potential adverse effects. Geneva, WHO, 2012.

World Health Organization. Effect of increased potassium intake on cardiovascular disease, coronary heart disease and stroke. Geneva, WHO, 2012.

**Supplementary Table 8:** **Non-dietary causes of chronic hyperkalemia in children**

Pseudohyperkalemia

Impaired renal excretion:

- Low GFR
- Medications:
  - Potassium sparing diuretics (spironolactone, triamterene, amiloride)
  - RASi (ACE inhibitors, ARBs, direct renin inhibitors)
  - Others (e.g. calcineurin inhibitors, NSAIDs, trimethoprim)
- Chronic metabolic acidosis
- Tubular disorders
  - Low aldosterone (e.g. Gordon syndrome)
  - Aldosterone resistance (e.g. pseudohypoaldosteronism)

Constipation

Impaired cellular entry of potassium (beta-blockers)

Exogenous K administration:

- Oral potassium supplements
- Potassium-containing medications (e.g. Penicillin V potassium)

Abbreviations: GFR, glomerular filtration rate; RASi, renin-angiotensin-aldosterone system inhibitors; ACE, angiotensin converting enzyme; ARBs, angiotensin receptor blockers; NSAIDs, nonsteroidal anti-inflammatory drugs.

**Supplementary Table 9: Non-dietary causes of chronic hypokalemia in children**

Dialysate K losses

Medications

- K-binding resins (e.g., sodium polystyrene sulfonate)

Diuretics (loop or thiazide)

Gastrointestinal K losses

- Vomiting or drainage from gastrostomy tubes
- Diarrhea
- Laxative or enema abuse

Renal tubular disorders (e.g. cystinosis, Bartter syndrome)

**Supplementary Table 10:** **Example of a diluted formula to lower potassium intake for an infant**

Standard whey dominant infant formula mixed as instructed by the manufacturer, 3 scoops (13 g) formula powder added to 90 ml water to make 100 ml formula.

|  | Energy (kcal) | Protein (g) | CHO (g) | Fat (g) | K (mg) | PO4 (mg) |
| --- | --- | --- | --- | --- | --- | --- |
| 3 scoops infant formula powder  (13 g) | 68 | 1.3 | 7.2 | 3.6 | 69 | 24 |

Diluted formula (32% reduction in K content) with added energy and protein modules to preserve energy and protein density.

|  | Energy (kcal) | Protein (g) | CHO (g) | Fat (g) | K (mg) | PO4 (mg) |
| --- | --- | --- | --- | --- | --- | --- |
| 2 scoops infant formula powder  (19 g) | 47 | 0.9 | 5.0 | 2.5 | 47 | 17 |
| 0.5 g protein powder | 2 | 0.5 | 0 | 0 | 0 | 0 |
| 2.5 ml fat emulsion | 11 | 0 | 0 | 1.3 | 0 | 0 |
| 2 g glucose polymer | 8 | 0 | 2.0 | 0 | 0 | 0 |
| + water up to  100 ml |  |  |  |  |  |  |
| per 100 ml | 68 | 1.4 | 7.0 | 3.8 | 47 | 17 |

A vitamin and mineral supplement (without vitamin A) is likely to be necessary to achieve nutritional adequacy.

A phosphate supplement may also be required.

**Supplementary Table 11: Pretreatment of liquids to reduce the potassium content**

| - Add sodium polystyrene sulfonate (SPS) to the bottle of formula or expressed breastmilk (EBM), allowing it to precipitate for 30 minutes in a refrigerator, then decant the formula/EBM from the residue that has settled in the bottom (47). - The dose of SPS should be titrated according to individual tolerance and serum K levels. - A starting dose of 0.4-1.5 g/100 ml or 0.25-1.0 g/mmol K has been suggested (41). |
| --- |

**Supplementary Table 12: Potassium content of foods per unit of fiber**

| <100mg K/g fiber | 100-200mg K/g fiber | >200mg K/g fiber |
| --- | --- | --- |
| Fruits |  |  |
| Raspberry, blueberry, apple, strawberry | Peach, grape, kiwi fruit, cherry, banana | Pineapple |
| Vegetables |  |  |
| Carrot, peas | Broccoli, beetroot, courgette (zucchini), onion, sweet potato | Cucumber, pumpkin, tomato (peeled) |
| Cereals (grains) |  |  |
| Cornflakes, whole bread, pasta, rice |  |  |

Source: Cupisti *et al*, 2018 (49). Potassium and fibre contents from the European Institute of Oncology Food Composition Database (personal communication).
